# Foxp1-mediated programming of limb-innervating motor neurons from mouse and human embryonic stem cells

**DOI:** 10.1038/ncomms7778

**Published:** 2015-04-14

**Authors:** Katrina L. Adams, David L. Rousso, Joy A. Umbach, Bennett G. Novitch

**Affiliations:** 1 Department of Neurobiology, David Geffen School of Medicine at UCLA, 610 Charles E Young Dr East, TLSB 3024, Los Angeles, California 90095, USA; 2 Eli and Edythe Broad Center of Regenerative Medicine and Stem Cell Research, University of California, Los Angeles, 610 Charles E Young Dr East, TLSB 3024, Los Angeles, California 90095, USA; 3 Molecular Biology Institute Graduate Program, University of California, Los Angeles, 610 Charles E Young Dr East, TLSB 3024, Los Angeles, California 90095, USA; 4 Department of Molecular and Medical Pharmacology, University of California, Los Angeles, 610 Charles E Young Dr East, TLSB 3024, Los Angeles, California 90095, USA

## Abstract

Spinal motor neurons (MNs) control diverse motor tasks including respiration, posture and locomotion that are disrupted by neurodegenerative diseases such as amyotrophic lateral sclerosis and spinal muscular atrophy. Methods directing MN differentiation from stem cells have been developed to enable disease modelling *in vitro*. However, most protocols produce only a limited subset of endogenous MN subtypes. Here we demonstrate that limb-innervating lateral motor column (LMC) MNs can be efficiently generated from mouse and human embryonic stem cells through manipulation of the transcription factor Foxp1. Foxp1-programmed MNs exhibit features of medial and lateral LMC MNs including expression of specific motor pool markers and axon guidance receptors. Importantly, they preferentially project axons towards limb muscle explants *in vitro* and distal limb muscles *in vivo* upon transplantation–hallmarks of *bona fide* LMC MNs. These results present an effective approach for generating specific MN populations from stem cells for studying MN development and disease.

## Abstract (web summary)

The differentiation of spinal motor neurons (MNs) from mouse and human embryonic stem cells provides opportunities to model MN development and disease, but most protocols produce only a subset of the MN subtypes found *in vivo*. Here the authors show that limb projecting lateral motor column MNs can be efficiently generated though the expression of Foxp1.

## Graphical abstract

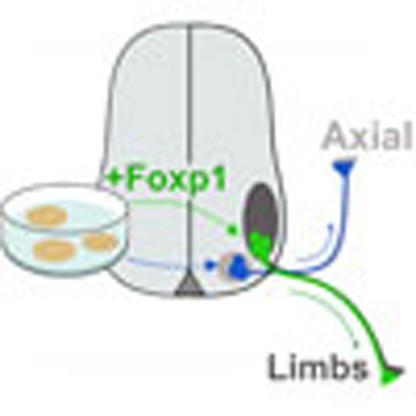

The ability to derive specialized cell types from pluripotent stem cells has allowed for novel insights into development and disease, particularly in the case of neurodegenerative diseases where specific neuronal populations are affected. To accurately model development and disease, stem cell-derived populations must fully recapitulate endogenous cell populations—both in the diversity of cell types generated and their functional behaviour. A prime example includes the differentiation of spinal motor neurons (MNs) from mouse and human embryonic stem cells (ESCs) and induced pluripotent stem cells (iPSCs), which has provided an unprecedented opportunity to model the pathogenesis of MN diseases such as amyotrophic lateral sclerosis (ALS) and spinal muscular atrophy^1^,^2^,^3^,^4^,^5^. However, despite these advances, *in vitro* MN differentiation protocols typically produce only a limited subset of endogenous MN populations^6^.

In developing mouse embryos, spinal MNs are organized into columns that innervate distinct muscle targets along the length of the body^7^,^8^,^9^. In addition to their muscle targets, motor columns are distinguished by their differential and combinatorial expression of LIM-homeodomain proteins, Hox proteins and other transcription factors^10^,^11^,^12^,^13^. The medial motor column (MMC) innervates axial muscles along the entire rostrocaudal axis. The lateral motor column (LMC) innervates limb muscles at cervical and lumbar levels. LMC MNs are further subdivided into medial (LMCm) and lateral (LMCl) populations that innervate the ventral and dorsal limb muscles, respectively. The hypaxial motor column (HMC) innervates respiratory muscles, including the diaphragm, intercostals and abdominal muscles at different levels of the spinal cord. Last, the preganglionic motor columns (PGC) innervate the sympathetic and parasympathetic ganglia and are present only at thoracic and sacral levels. The generation of these different MN subtypes and their subsequent assembly into motor circuits depends on the expression and function of key fate determinants in differentiating and postmitotic MNs^7^,^8^,^9^,^10^,^11^,^12^,^13^.

Most ESC to MN differentiation protocols rely on the use of retinoic acid (RA) and Sonic hedgehog (Shh) or Shh pathway agonists to mimic the natural process of MN formation *in vivo*^14^,^15^,^16^,^17^. However, these protocols appear to introduce significant bias in the formation of only select MN subtypes, predominantly MMC, with limited production of LMC MNs^6^,^15^. Spontaneous and RA-independent differentiation of ESCs have been shown to increase the diversity of MNs generated to an extent, but the efficiency of MN production tends to be reduced and few LMCl-like MNs form^18^,^19^. An alternative approach is to directly programme MN subtypes, taking advantage of our knowledge of the transcriptional programmes that specify different MN fates. One of the major fate determinants of LMC MNs *in vivo* is the Forkhead domain transcription factor Foxp1. All LMC and PGC MNs in the developing spinal cord express Foxp1, and *Foxp1*-mutant animals have severely reduced innervation and motor control of limb muscles^12^,^13^,^20^. We thus examined whether Foxp1 function could be used to direct the differentiation of limb-innervating MNs from ESCs, without compromising differentiation efficiency.

In this study, we demonstrate that Foxp1 misexpression in differentiating MNs suffices to generate *bona fide* LMC MNs in the mouse spinal cord and both mouse and human ESC-derived MNs *in vitro*. These Foxp1-programmed ESC-derived MNs express molecular markers of LMCm and LMCl motor columns and specific LMC motor pools, indicating conversion from an MMC to LMC fate. Foxp1-programmed MNs express LMC axon guidance receptors and preferentially project axons towards limb muscle explants *in vitro*, as well as distal limb muscles in chick embryos after transplantation. Last, Foxp1-programmed MNs preferentially form synapses with limb muscles *in vitro* and *in vivo* in contrast to control MMC-like MNs, which preferentially innervate axial muscles. Together, these results illustrate the feasibility of generating specific subtypes of MNs from pluripotent stem cells using a transcriptional programming approach, and the importance of MN diversity for achieving functionally distinct behaviours.

## Results

### Foxp1 misexpression restores LMC production in *Foxp1* mutants

Previous work in our laboratory and others has shown that Foxp1 is necessary and sufficient for the generation and function of LMC and PGC MNs in the mouse spinal cord^12^,^13^,^20^. In *Foxp1* mutants, LMC and PGC MNs transform into MMC and HMC MNs, illustrated by changes in molecular markers, settling position within the ventral horn of the spinal cord and axon projections^12^,^13^. Accordingly, *Olig2::Cre; Foxp1*^*fl/fl*^ mutants, in which Foxp1 is removed from MN progenitors, are unable to move their forelimb and hindlimb muscles due to the inability of MNs to coalesce into functional motor pools needed to form sensory-motor circuits^20^. In contrast, *Hb9::Foxp1* transgenic mice, in which Foxp1 is misexpressed in most spinal MNs under the Hb9 promoter, display an increased generation of LMC and PGC MNs, and a corresponding decrease in MMC and HMC populations^12^,^13^.

**Figure 1:**
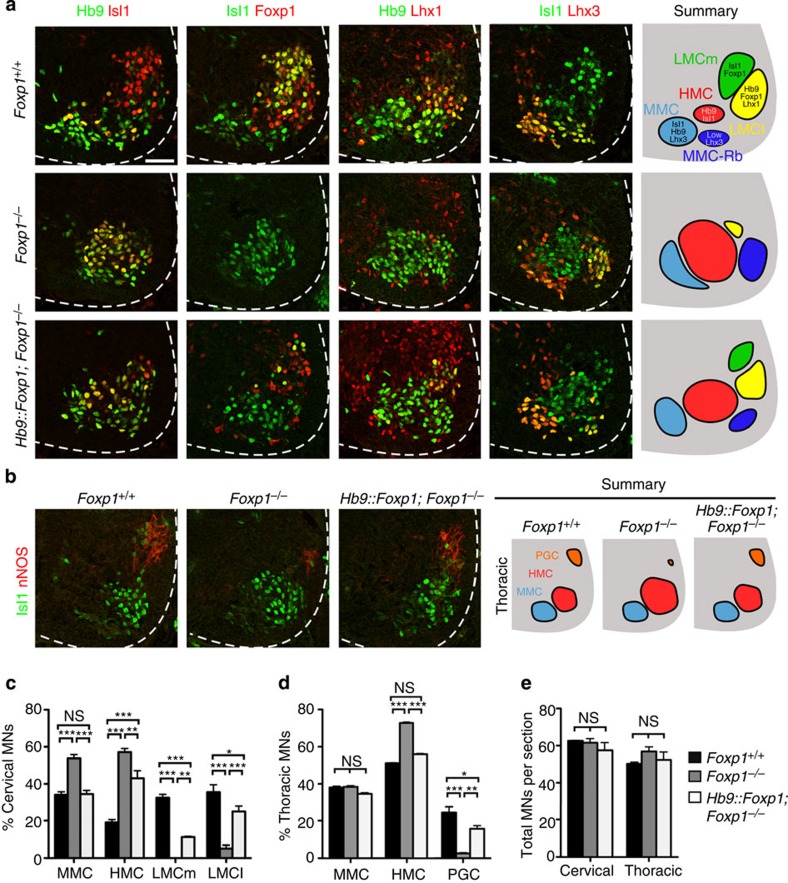
Foxp1 misexpression rescues the *Foxp1*-mutant phenotype. Analysis of MN subtypes in the ventral horn of E12.5 *Foxp*^*+/+*^, *Foxp*^*−/−*^ and *Hb9::Foxp1; Foxp1*^*−/−*^ littermate embryo spinal cords. Scale bar, 50 μm. (**a**) MN subtypes at cervical levels. *Foxp1*^*−/−*^ embryos had reduced numbers of LMCm (Isl1^+^/Foxp1^+^) and LMCl (Hb9^+^/Lhx1^+^) MNs, while HMC (Hb9^+^/Isl1^+^) and MMC-rhomboideus (Isl1^+^/Lhx3^low^) MNs were expanded compared with *Foxp1*^*+/+*^ embryos^13^. *Hb9::Foxp1; Foxp1*^*−/−*^ embryos had partially restored LMCm and LMCl MNs that resided at the correct lateral positions, and reduced numbers of ectopic HMC and MMC-Rb MNs, compared with *Foxp1*^*−/−*^ embryos. Rb, rhomboideus. (**b**) MN subtypes at thoracic levels. The loss of PGC (Isl1^+^/nNOS^+^) MNs in *Foxp1*^*−/−*^ embryos was rescued in *Hb9::Foxp1; Foxp1*^*−/−*^ embryos. (**c**) Quantification of MN percentages at cervical levels (mean±s.e.m.; *n*=3 sections averaged per embryo, 3 embryos per genotype; two-way analysis of variance (ANOVA) with Bonferroni adjustment). MMC MNs: ***P<0.0001. HMC MNs: ***P<0.0001 and **P=0.001. LMCm MNs: ***P<0.0001 and **P=0.006. LMCl MNs: ***P<0.0001 and *P=0.01. NS, not significant, *P*>0.5. (**d**) Quantification of MN percentages at thoracic levels (mean±s.e.m.; *n*=3 sections averaged per embryo, 3 embryos per genotype; two-way ANOVA with Bonferroni adjustment). HMC MNs: ***P<0.0001. PGC MNs: ***P<0.0001, **P=0.0001 and *P=0.0023. NS, not significant, *P*>0.5. (**e**) Quantification of total number of MNs per section (mean±s.e.m.; *n*=3 sections averaged per embryo, 3 embryos per genotype; two-way ANOVA with Bonferroni adjustment). NS, not significant. *P*>0.5.

To test whether the *Hb9::Foxp1* transgene could direct LMC MN formation d*e novo*, we interbred *Hb9::Foxp1* mice with *Foxp1*^*−/−*^ mice and analysed MN formation in different mutant and transgenic allele combinations (Fig. 1). At E12.5, three distinct populations of MNs: MMC, HMC and LMC were present in the cervical spinal cord of both *Foxp1*^*+/+*^ and *Foxp1*^*+/−*^ embryos (Fig. 1a)^12^,^13^. As previously described, *Foxp1*^*−/−*^ embryos showed an almost complete loss of LMC MNs, and reciprocal expansion of Hb9^+^/Isl1^+^/Lhx3^*−*^ HMC MNs and Isl1^+^/Lhx3^+^ MMC-like MNs (Fig. 1a)^12^,^13^. LMC MN formation was significantly restored in *Hb9::Foxp1*; *Foxp1*^*−/−*^ embryos, with concomitant reductions in both ectopic HMC and MMC MNs (Fig. 1a,c). A similar trend was observed in the thoracic spinal cord (Fig. 1b). PGC MNs—another Foxp1-dependent population—returned in *Hb9::Foxp1*; *Foxp1*^*−/−*^ embryos, coincident with a nearly complete suppression of ectopic HMC MNs (Fig. 1d). There was no significant difference in total MN number between all three genotypes, indicating that changes in MN subtypes were likely a result of fate conversion rather than variations in cell death and survival (Fig. 1e). Overall, these results indicate that transgenic Foxp1 expression is sufficient to generate LMC and PGC MNs and suppress ectopic MMC and HMC MN formation in a *Foxp1*-null background *in vivo*.

### Foxp1-programmed ESC MNs form LMCm and LMCl subtypes

**Figure 2:**
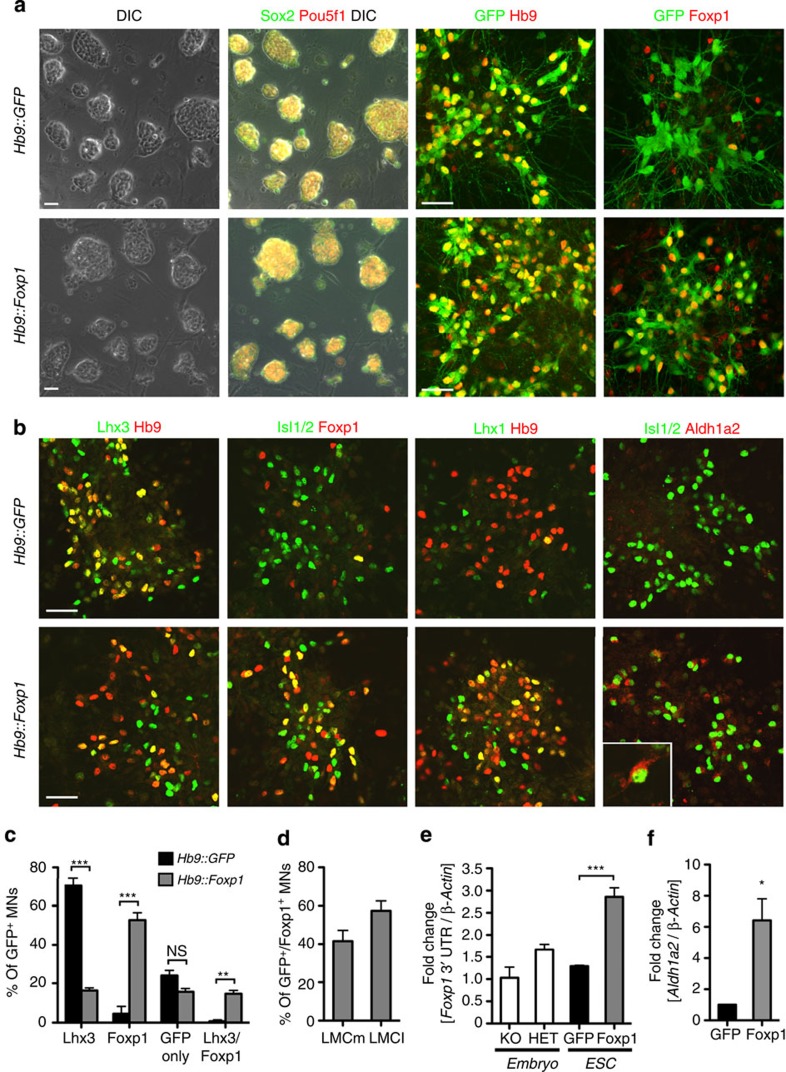
*Hb9::Foxp1* ESCs generate LMCm and LMCl MNs. (**a**) Newly derived ESC lines displayed characteristic ESC morphology and expressed pluripotency markers. Both *Hb9::GFP* and *Hb9::Foxp1* ESC lines differentiated into GFP^+^/Hb9^+^ MNs, but only *Hb9::Foxp1* ESC-derived MNs expressed high levels of Foxp1 protein. Scale bars, 50 μm. (**b**) Analysis of MN columnar marker expression in ESC-derived MNs. The majority of control *Hb9::GFP* ESC-derived MNs expressed Lhx3 and low levels of Foxp1, Lhx1 and Aldh1a2. *Hb9::Foxp1* ESC-derived MNs expressed reduced levels of Lhx3 and increased levels of LMCm (Isl1^+^/Foxp1^+^) and LMCl (Hb9^+^/Lhx1^+^) MN markers. Inset shows a single Aldh1a2^+^ MN. Scale bars, 50 μm. (**c**) Quantification of MN subtypes generated by each ESC line (mean±s.e.m.; *n*=3 independent experiments, 373 *Hb9::GFP* MNs and 403 *Hb9::Foxp1* MNs total; Student’s *t*-test). Lhx3: ***P=0.0002. Foxp1: ***P=0.0008. Lhx3/Foxp1: **P=0.002. NS, not significant, *P*>0.5. (**d**) Quantification of the percentages of *Hb9::Foxp1* ESC-derived Foxp1^+^ MNs that express markers of LMCm (GFP^+^/Foxp1^+^) and LMCl (GFP^+^/Foxp1^+^/Lhx1^+^) MNs (mean±s.e.m.; *n*=3 independent experiments, 222 *Hb9::Foxp1* MNs total). (**e**) Endogenous Foxp1 mRNA was expressed approximately threefold higher in *Hb9::Foxp1* ESC-derived MNs, compared with *Hb9::GFP* ESC-derived MNs. E11.5 *Foxp1*^*+/−*^ and *Foxp1*^*−/−*^ purified MNs were used as controls (mean±s.e.m.; *n*=3 *Foxp1*^*+/−*^ embryos, 3 *Foxp1*^*−/−*^ embryos, 3 independent RNA collections of *Hb9::GFP* and *Hb9::Foxp1* MNs; one-way analysis of variance with Bonferroni adjustment). ***P=0.001. (**f**) *Aldh1a2* mRNA levels were elevated approximately sixfold higher in *Hb9::Foxp1* ESC-derived MNs, compared with *Hb9::GFP* controls (mean±s.e.m.; *n*=5 independent RNA collections of *Hb9::GFP* and *Hb9::Foxp1* MNs; paired two-tailed *t*-test). *P=0.017.

The ability of the *Hb9::Foxp1* transgene to rescue *Foxp1*^*−/−*^ MN phenotypes *in vivo* led us to hypothesize that Foxp1 misexpression during ESC differentiation would be sufficient to generate LMC MNs *in vitro*. Foxp1 overexpression in neural progenitors disrupts MN differentiation, mimicking Foxp4 overexpression phenotypes^21^; highlighting the importance of overexpressing Foxp1 only in postmitotic MNs. Therefore, we derived mouse ESC lines from *Hb9::Foxp1; Hb9::*Green Fluorescent Protein *(GFP)* blastocysts. Four *Hb9::Foxp1; Hb9::GFP* (hereafter termed *Hb9::Foxp1*) ESC lines were generated, and each exhibited characteristic ESC colony appearance and pluripotency marker expression (Fig. 2a). The widely used HBG3 *Hb9::GFP* ESC line^15^, as well as newly created isogenic *Hb9::GFP* ESC lines, were used as controls for comparison throughout this study. All *Hb9::GFP* ESC lines behaved equivalently. *Hb9::GFP* and *Hb9::Foxp1* ESC lines generated similar numbers of GFP^+^/Hb9^+^ MNs, indicating that Foxp1 misexpression did not affect MN production efficiency (Fig. 2a). On average, 40% of all ESC-derived cells expressed GFP, as determined by immunohistochemistry and fluorescence-activated cell sorting (FACS) analysis. *Hb9::Foxp1* ESC-derived MNs expressed 15- to 20-fold higher levels of *Foxp1a* coding messenger RNA (mRNA), compared with all *Hb9::GFP* control MNs. *Hb9::Foxp1* ESC-derived MNs also showed a 2.86±0.02-fold increase (mean±s.e.m.) in levels of *Foxp1* 3′ untranslated region (UTR) mRNA expression compared with *Hb9::GFP* ESC-derived MNs (Fig. 2e), indicating activation of the endogenous *Foxp1* locus. *Foxp1* 3′UTR mRNA levels were also reduced in *Foxp1*^*−/−*^ embryonic MNs compared with *Foxp1*^*+/−*^ embryos (Fig. 2e). These data together suggest that Foxp1 promotes its own expression through a positive feedback loop.

We next analysed the ESC-derived MNs for specific motor column markers to determine whether Foxp1 misexpression alters MN subtype formation *in vitro*. In agreement with previously published results^6^,^15^, the majority (70.5±3.8% mean±s.e.m.) of control *Hb9::GFP* ESC-derived MNs expressed Lhx3, a marker of MMC MNs (Fig. 2b). Only 4.6±3.7% of *Hb9::GFP* MNs expressed LMC markers (Foxp1, Lhx1 and Aldh1a2) 7 days after addition of RA and Smoothened agonist (SAG) (Fig. 2b,c). In contrast, *Hb9::Foxp1* ESC-derived MNs displayed significantly reduced numbers of Lhx3^+^ cells (16.5±1.4%) and increased numbers of cells positive for LMCm (Isl1/Foxp1) and LMCl (Hb9/Lhx1) markers (52.78±3.8% were Foxp1^+^, and of these 57.4±5.2% expressed Lhx1 while 41.6±5.6% expressed Isl1) (Fig. 2b,c). Interestingly, 14.8±1.8% of *Hb9::Foxp1* ESC-derived MNs expressed both Lhx3 and Foxp1, a cell population that is rarely found *in vivo*. However, this population disappeared with additional time in culture (Supplementary Fig. 1a,b), likely due to resolution in cell fates associated with the cross-repressive activities of Lhx3 and Foxp1 (refs 12, 13). We did not observe any changes in the numbers of apoptotic cells assessed by activated Caspase3 antibody staining or total MN numbers over the time in culture (Supplementary Fig. 1c,d).

We also examined ESC-derived MNs for expression of Aldh1a2, an RA-synthesizing enzyme expressed by LMC MNs *in vivo*^22^. *Aldh1a2* mRNA levels were 6.4±1.4-fold higher in *Hb9::Foxp1* ESC-derived MNs compared with *Hb9::GFP* controls (Fig. 2b,f). Within the LMC-like MNs produced by the *Hb9::Foxp1* ESCs, 57.4±5.2% (mean±s.e.m.) expressed Lhx1, suggesting a slight bias in LMCl formation over LMCm (Fig. 2d). This could reflect the presence of RA in the differentiation media, seeing as LMCl formation *in vivo* is influenced by retinoid signalling^22^. Consistent with this possibility, altering RA levels in the differentiation protocol led to corresponding changes in Lhx1^+^ *Hb9::Foxp1* MNs (Supplementary Fig. 2). Together, these results indicate that Foxp1 misexpression is sufficient to transform the molecular identity of ESC-derived MNs from an MMC phenotype to an LMC phenotype.

### Foxp1-programmed MNs express LMC motor pool markers

**Figure 3:**
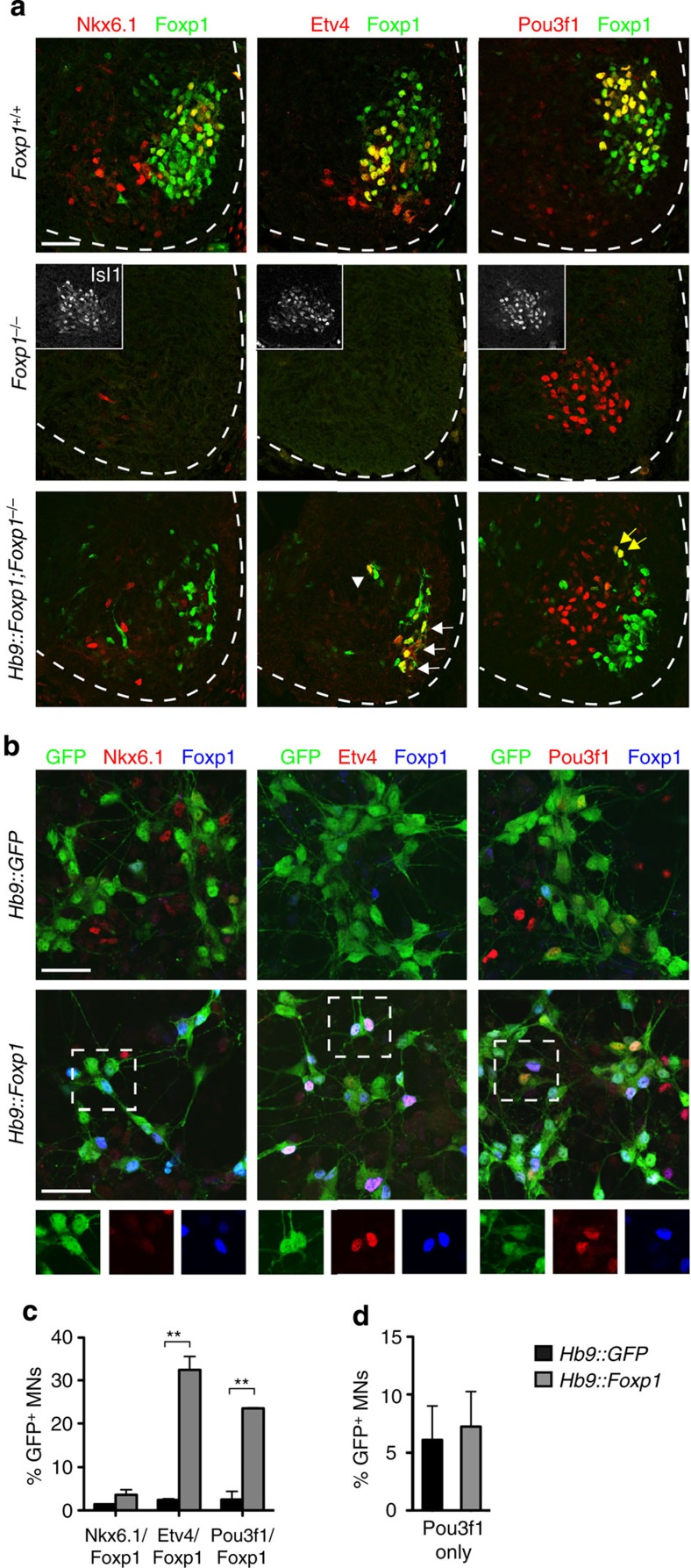
Foxp1 misexpression induces LMC motor pool markers. (**a**) Analysis of cervical LMC motor pools in E12.5 *Foxp1*^*+/+*^, *Foxp1*^*−/−*^ and *Hb9::Foxp1*; *Foxp1*^*−/−*^ embryos. All LMC motor pools were lost in *Foxp1*^*−/−*^ embryos. The Pou3f1^+^ MNs in *Foxp1*^*−/−*^ embryos represent the expanded HMC MN population (identified by their medial position). Insets depict Islet1 co-staining of the same section. The Etv4 motor pool was restored in *Hb9::Foxp1; Foxp1*^*−/−*^ embryos (white arrows), with some Foxp1^+^/Etv4^+^ MNs located more dorsally (arrowhead). The Nkx6.1 and Pou3f1 motor pools were not restored in *Hb9::Foxp1; Foxp1*^*−/−*^ embryos, although a small number of Foxp1^+^/Pou3f1^+^ MNs were identified (yellow arrows). Scale bar, 50 μm. (**b**) Control *Hb9::GFP* ESC-derived MNs did not express any LMC motor pool markers. *Hb9::Foxp1* ESC-derived MNs expressed Etv4 and Pou3f1 at high levels, but did not express Nkx6.1. Boxed regions are depicted as single-colour images below each image. Scale bars, 50 μm. (**c**) Quantification of motor pool markers in ESC-derived MNs (mean±s.e.m.; *n*=2 independent experiments, 938 *Hb9::GFP* MNs and 723 *Hb9::Foxp1* MNs total; Student’s *t*-test). Etv4: **P=0.009. Pou3f1: **P=0.008. (**d**) Foxp1 misexpression did not alter the percentage of Pou3f1^+^/Foxp1^*−*^ ESC-derived HMC-like MNs (mean±s.e.m.; *n*=3 independent experiments, 390 *Hb9::GFP* MNs and 255 *Hb9::Foxp1* MNs total).

In the embryo, LMC MNs are further divided into individual motor pools, MN groups that innervate the same muscle. At cervical regions, these pools include Etv4^+^ (also known as Pea3) LMCl and LMCm pools that innervate the latissimus dorsi and cutaneous maximus muscles^23^,^24^; Nkx6.1^+^ LMCm pools that innervate the biceps and other forearm flexor muscles; and a Pou3f1^+^ (also known as SCIP) LMCm pool that innervates the flexor carpi ulnaris muscle of the lower forearm (Fig. 3a)^24^,^25^. *In vivo*, the expression of these pool markers is initiated by exposure of MN growth cones to signals emanating from the surrounding peripheral tissue and developing muscles. For example, Etv4 is expressed in LMC MNs on exposure to glia-derived neurotrophic factor (GDNF) as LMC MN axons approach the developing limbs^26^. In *Foxp1*^*−/−*^ embryos, there is a complete loss of Etv4^+^ and Nkx6.1^+^ LMC motor pools at cervical levels, and a conversion of Pou3f1^+^ LMC MNs to Pou3f1^+^ HMC MNs (Fig. 3a)^12^,^13^. To test whether Foxp1 misexpression in MNs could rescue these lost LMC motor pools, we analysed *Hb9::Foxp1; Foxp1*^*−/−*^ embryos for expression of pool markers. Interestingly, Etv4^+^ motor pools were significantly restored, while Nkx6.1^+^ and Pou3f1^+^ LMC motor pools were not (Fig. 3a). This deficiency may be attributed to the fact that both Nkx6.1^+^ and Pou3f1^+^ LMC motor pools are associated with the LMCm, which was only partially restored in *Hb9::Foxp1; Foxp1*^*−/−*^ embryos (Fig. 1a,c).

To determine whether Foxp1 misexpression results in LMC motor pool formation *in vitro*, we analysed *Hb9::GFP* and *Hb9::Foxp1* ESC-derived MNs for these same pool markers. Control *Hb9::GFP* ESC-derived MNs did not express Etv4, Nkx6.1 or Pou3f1 on day 7 of differentiation, despite the presence of GDNF in the culture medium (Fig. 3b). Conversely, *Hb9::Foxp1* ESC-derived MNs expressed both Etv4 and Pou3f1 (mean±s.e.m.=32.4±3.1% and 23.5±0.14% of GFP^+^ MNs, respectively), but did not express Nkx6.1 despite the clear presence of LMCm MNs in the culture (Fig. 3b). The factor(s) required to generate Nkx6.1^+^ LMC motor pools are not known, and we surmise that such factors are likely missing from the culture media. To determine whether the addition of neurotrophic factors to the MN cultures biases the types of motor pools generated, we analysed *Hb9::Foxp1* ESC-derived MNs cultured with and without GDNF. GDNF removal reduced the numbers of Etv4^+^ MNs formed, but did not affect expression of other pool markers (Supplementary Fig. 3a,b). We also confirmed that Pou3f1^+^/Foxp1^+^ MNs did not express Lhx1, indicating an LMCm phenotype. Etv4^+^/Foxp1^+^ MNs were more complex, with some cells expressing and others lacking Lhx1, suggesting the formation of both LMCl and LMCm pools (Supplementary Fig. 3c). A small number of Pou3f1^+^/Foxp1^*−*^ GFP^+^ MNs were also identified in both *Hb9::GFP* and *Hb9::Foxp1* ESC-derived MN cultures, which likely represent HMC MNs (Fig. 3d)^13^,^27^,^28^.

We next examined whether Foxp1 misexpression had an impact on Hox proteins, which are implicated in both columnar and pool identities^25^,^29^, but did not observe any significant differences in the cultures. The majority of both *Hb9::GFP* and *Hb9::Foxp1* ESC-derived MNs expressed Hoxa5 and Hoxc6, which are characteristic of rostral forelimb MNs, and did not express Hoxc8 or Hoxa9 associated with more caudal MN subtypes (Supplementary Fig. 4). These data indicate that the ability of Foxp1 misexpression to convey LMC motor pool identities is most likely independent of changes in Hox activities.

**Figure 4:**
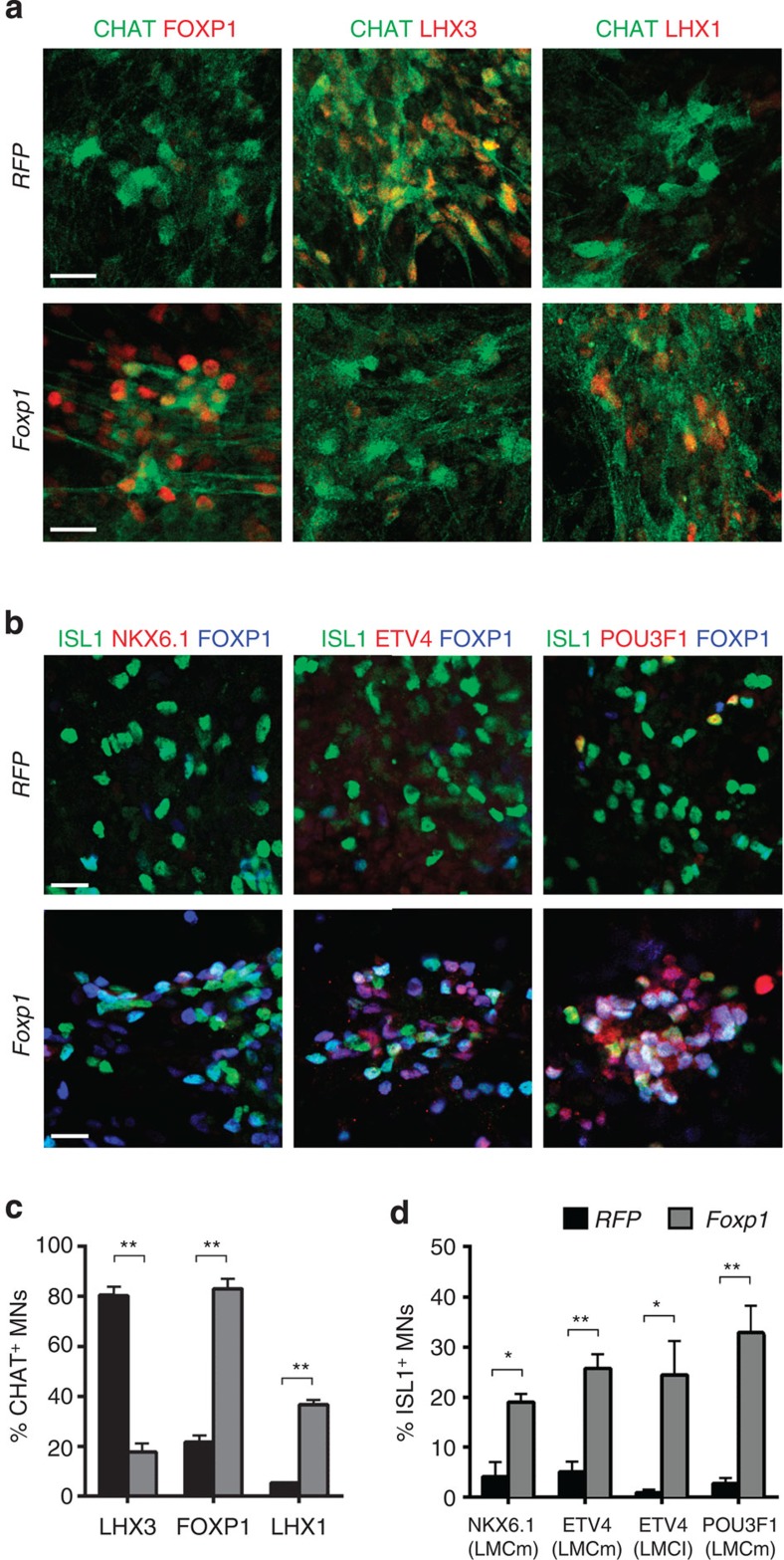
FOXP1 promotes LMC MN formation from human ESCs. (**a**) Control RFP-transduced human ESC-derived MNs (identified by CHAT expression) expressed high levels of LHX3 and low levels of FOXP1 and LHX1. FOXP1-transduced human ESC-derived MNs expressed increased levels of LHX1 and decreased levels of LHX3. (**b**) Control RFP-transduced human ESC-derived MNs did not express high levels of known LMC motor pool markers such as ETV4, POU3F1 and NKX6.1. By contrast, many FOXP1-transduced human ESC-derived MNs expressed ETV4 or POU3F1, with a smaller number expressing NKX6.1. (**c**) Quantification of MN subtype markers in transduced human ESC-derived MNs (mean±s.e.m.; *n*=2 independent experiments, 700 RFP-transduced MNs and 655 FOXP1-transduced MNs total; Student’s *t*-test). LHX3: **P=0.0055. FOXP1: **P=0.0056. LHX1: **P=0.0035. (**d**) Quantification of LMC motor pool markers in transduced human ESC-derived MNs (mean±s.e.m.; *n*=3 independent experiments, 2342 RFP-transduced MNs and 1624 FOXP1-transduced MNs total; Student’s *t*-test). NKX6.1: *P=0.0108. ETV4 (LMCm): **P=0.0042. ETV4 (LMCl): *P=0.0263. POU3F1: **P=0.0053. NKX6.1, ETV4 (LMCm) and POU3F1 motor pools were identified by co-localization of ISL1, FOXP1 and the respective motor pool marker. The ETV4 (LMCl) motor pool was identified by co-localization of FOXP1 and ETV4.

Last, we tested whether Foxp1 misexpression could similarly enhance LMC MN production from human ESCs. H9 ESCs were first differentiated into MNs and then transduced with lentiviruses encoding red fluorescent protein (RFP) alone or together with the human *FOXP1* gene. MNs infected with the RFP-only virus predominantly expressed the MMC marker LHX3 along with general MN differentiation markers such as ISL1 and choline acetyltransferase (CHAT) (Fig. 4a,c). A small population of endogenous LMC-like FOXP1^+^ CHAT^+^ MNs was detectable under these control cultures, though LHX1^+^ MNs were scarce (Fig. 4a,c). However, when human ESC-derived MNs were infected with the virus containing FOXP1, LHX3 expression declined while LHX1 levels became readily detectable (Fig. 4a,c). These FOXP1-transduced MNs further expressed LMC motor pool markers including ETV4, POU3F1 and NKX6.1 (Fig. 4b,d), recapitulating the results seen with the misexpression of Foxp1 in mouse ESC-derived MNs. Thus, Foxp1 expression appears to be able to impart an LMC identity to ESC-derived MNs in human cells as well.

### Foxp1-programmed MNs preferentially project to limb muscles

**Figure 5:**
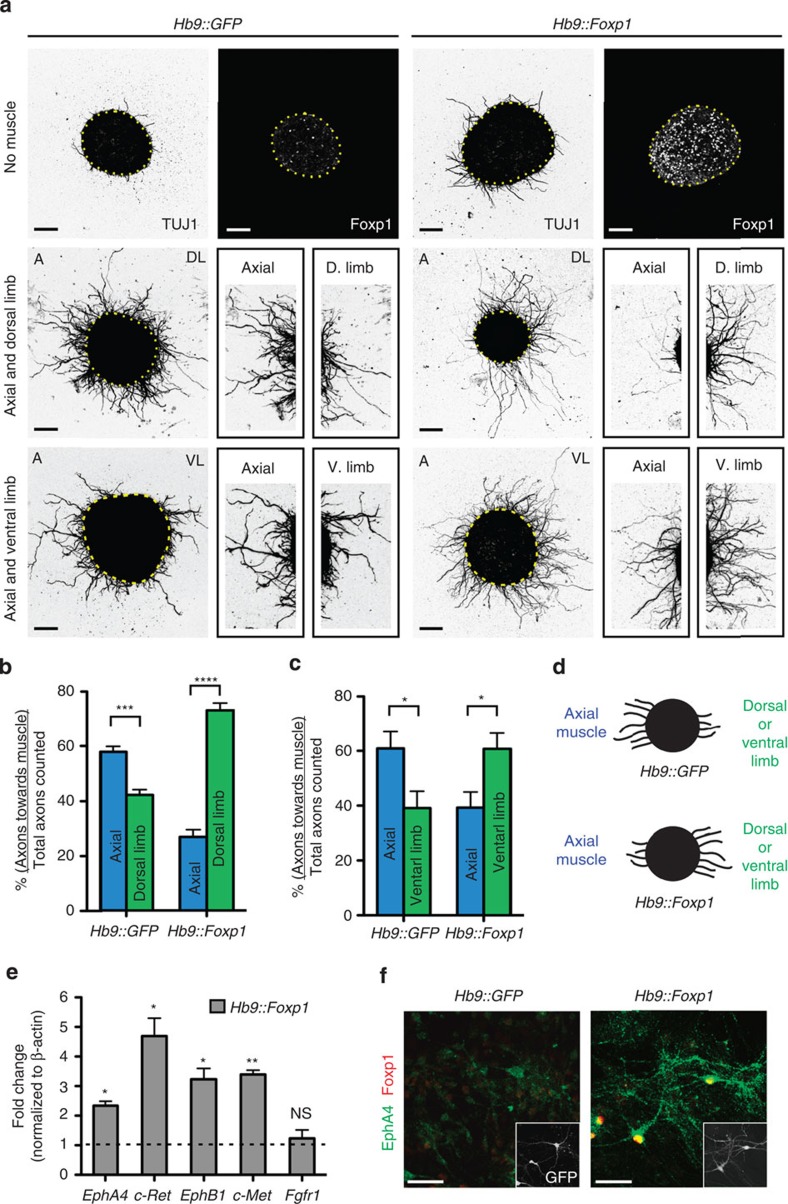
*Hb9::Foxp1* MNs project axons towards limb muscle explants *in vitro*. (**a**) Three-dimensional collagen cultures of EBs and different chick muscle explants. *Hb9::GFP* EBs projected more axons towards axial muscle than dorsal or ventral limb muscle. *Hb9::Foxp1* EBs projected more axons towards dorsal and ventral limb muscle than axial muscle explants. Scale bars, 100 μm. ‘A’=axial muscle. ‘DL’ and ‘D. limb’=dorsal limb muscle. ‘VL’ and ‘V. limb’=ventral limb muscle. (**b**) Quantification of *Hb9::GFP* and *Hb9::Foxp1* axon projections when cultured between axial and dorsal limb explants (mean±s.e.m.; *n*=5 cultures per cell line; Student’s *t*-test). ***P=0.0005. ****P<0.0001. (**c**) Quantification of *Hb9::GFP* and *Hb9::Foxp1* axon projections when cultured between axial and ventral limb explants (mean±s.e.m.; *n*=5 cultures per cell line; Student’s *t*-test). *P=0.0465 for *Hb9::GFP* and *P=0.0386 for *Hb9::Foxp1*. (**d**) Schematic summary of muscle explant results. (**e**) *Hb9::Foxp1* ESC-derived MNs expressed increased levels of *EphA4*, *c-Ret*, *EphB1* and *c-Met* mRNA, compared with *Hb9::GFP* ESC-derived MNs. There was no change in *Fgfr1* expression (mean±s.e.m.; *n*=3 independent RNA collections of *Hb9::GFP* and *Hb9::Foxp1* MNs; paired two-tailed *t*-test). *P=0.0115 for *EphA4*, *P=0.0264 for *EphB1*, *P=0.0258 for *c-Ret*, **P=0.0037 for *c-Met*. NS, not significant, *P*>0.5. (**f**) *Hb9::Foxp1* ESC-derived MNs expressed higher levels of EphA4 compared with control *Hb9::GFP* ESC-derived MNs. Insets depict GFP staining of ESC-derived MNs. Scale bars, 50 μm.

It has been previously shown that ESC-derived MNs do not stably innervate developing limb muscles likely due to their lack of LMC character^6^. To determine whether Foxp1 misexpression in ESC-derived MNs alters axon projections, we embedded GFP^+^ embryoid bodies (EBs) in three-dimensional collagen beds between different chick embryo muscle explants and analysed axon outgrowth (Supplementary Fig. 5a). When *Hb9::GFP* and *Hb9::Foxp1* EBs were cultured alone, very few axons projected into the surrounding collagen and most were very short in length (Fig. 5a). However, when *Hb9::GFP* EBs were cultured between axial and dorsal hindlimb muscle explants, the majority of axons projected towards the axial muscle (mean±s.e.m.=56.4±1.8% axial versus 43.6±1.8% dorsal limb) (Fig. 5b,c). This trend was also seen when *Hb9::GFP* EBs were cultured between axial and ventral hindlimb muscle explants (mean±s.e.m.=60.9±6.2% axial versus 39.1±6.2% ventral limb) (Fig. 5b,c). In contrast, a significant percentage of *Hb9::Foxp1* EB axons projected towards dorsal hindlimb muscle (mean±s.e.m.=26.8±2.7% axial versus 73.2±2.7% dorsal limb) or ventral hindlimb muscle (mean±s.e.m.=39.3±5.8% axial versus 60.7±5.8% ventral limb) when cultured together with axial muscle explants (Fig. 5a–d). Similar results were observed when *Hb9::Foxp1* EBs were cultured between axial and forelimb muscle explants (mean±s.e.m.=36.1±3.4% axial versus 63.9±3.4% forelimb), indicating that *Hb9::Foxp1* motor axons were comparably attracted to both forelimb and hindlimb muscles (Supplementary Fig. 5b,c).

These differences in axon projections suggest changes in MN detection of guidance and/or outgrowth cues present in the muscle explants. We therefore analysed control *Hb9::GFP* and *Hb9::Foxp1* ESC-derived MNs for expression of known LMC axon guidance receptors. *Hb9::Foxp1* MNs expressed increased levels of the EphA4 and *c-Ret* receptors, which are highly expressed by LMCl MNs and critical for dorsal limb innervation *in vivo* (Fig. 5e,f)^30^,^31^,^32^. *Hb9::Foxp1* MNs also expressed increased levels of *EphB1*, which is expressed by embryonic LMCm MNs and important for ventral limb innervation (Fig. 5e)^33^. In addition, *Hb9::Foxp1* MNs expressed increased levels of *c-Met*, which is critical for both dorsal and ventral limb innervation (Fig. 5e)^34^,^35^. However, there was no significant difference in *Fgfr1* levels, which are important for MMC projections towards developing axial muscles^36^, possibly due to the residual population of Lhx3^+^ MNs present in *Hb9::Foxp1* MN cultures (Fig. 5e). Together, these results provide evidence that Foxp1 misexpression in ESC-derived MNs alters axon projections *in vitro* to favour innervation of limb muscles over axial muscles, most likely due to increased expression of LMC axon guidance receptors. It is important to note that while increased levels of EphA4 and EphB1 are consistent with a switch to an LMC identity, these molecules are not likely to be responsible for the axon projections towards limb muscle explants *in vitro*. These receptors and ligands are membrane bound^37^ and would therefore not diffuse through the collagen bed. We surmise that diffusible guidance cues, such as HGF and GDNF, likely direct *Hb9::Foxp1* MNs towards limb muscles in the *in vitro* setting.

### Foxp1-programmed MNs form synapses with limb muscles *in vitro*

**Figure 6:**
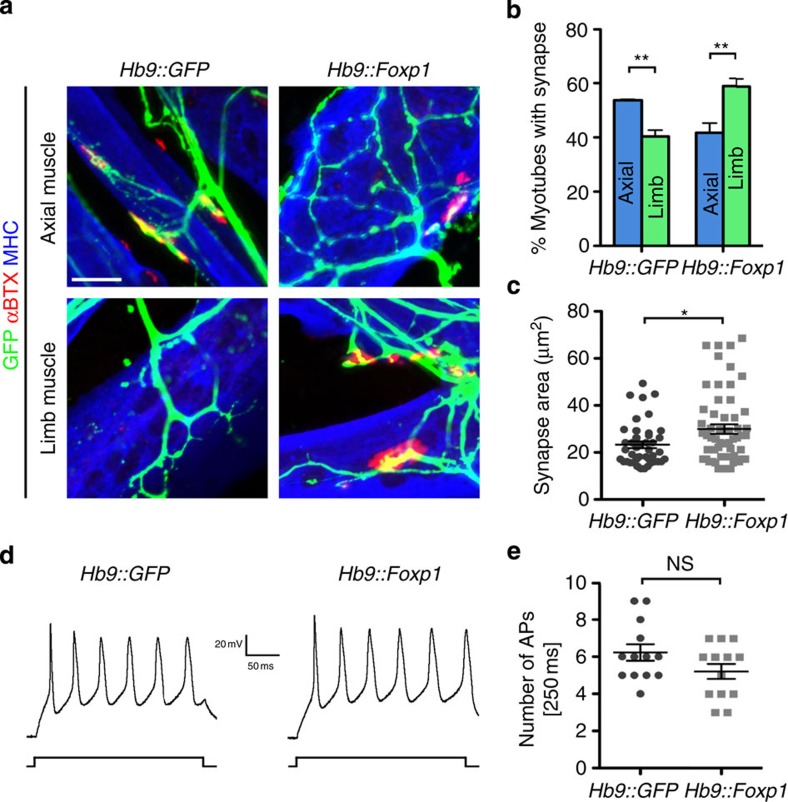
*Hb9::Foxp1* MNs preferentially form synapses with limb muscles. (**a**) *Hb9::GFP* MNs formed neuromuscular junctions (GFP^+^/αBTX^+^) with axial myotubes more frequently than with limb myotubes. By contrast, *Hb9::Foxp1* MNs formed synapses with limb-derived myotubes more frequently than with axial myotubes. Scale bar, 20 μm. (**b**) Quantification of the frequency of synapse formation with primary myotubes *in vitro*. The percentage of myotubes with GFP^+^/αBTX^+^ synapses over the total number of myotubes with GFP^+^ axon contacts was calculated for each cell line/muscle pairing (mean±s.e.m.; *n*=3 independent experiments, 20 images per experiment; Student’s *t*-test). **P=0.0046 for *Hb9::GFP* and **P=0.0081 for *Hb9::Foxp1*. (**c**) *Hb9::Foxp1* MNs formed significantly larger neuromuscular junctions with limb muscle than *Hb9::GFP* MNs, as determined by measuring the area of GFP^+^/αBTX^+^ synapses (mean±s.e.m.; *n*=5 images per cell line, 117 total synapses; Student’s *t*-test). *P=0.0143. (**d**) Representative recordings of *Hb9::GFP* and *Hb9::Foxp1* ESC-derived MNs. Both cell types responded to a +0.1 nA long duration (250 ms) current pulse with multiple action potentials. (**e**) Quantification of the number of action potentials (APs) fired by ESC-derived MNs during a +0.1 nA, 250 ms current pulse (mean±s.e.m.; *n*=13 cells per cell line; Student’s *t*-test). NS, not significant, *P*>0.5.

To determine whether these changes in muscle preference were also observed at the neuromuscular junction level, we generated ESC-derived MN and muscle co-cultures. Axial and dorsal limb muscle from chick embryos were dissociated and cultured as single myotubes for several days prior to the addition of ESC-derived MNs. Cultures were analysed for synapse formation using Alexa^647^-conjugated alpha-bungarotoxin (αBTX) to label neuromuscular junctions (Fig. 6a). GFP^+^/αBTX^+^ synapses were identified in all cultures, regardless of muscle type. To analyse the frequency of synapse formation with different muscle types, we calculated the percentage of myotubes with at least one GFP^+^/αBTX^+^ synapse, over the total number of myotubes contacted by GFP^+^ axons. Control *Hb9::GFP* MNs plated on axial myotubes resulted in a higher percentage (mean±s.e.m.=53.7±0.3%) of myotubes with GFP^+^/αBTX^+^ synapses, compared with limb myotube co-cultures (mean±s.e.m.=40.4±2.3%) (Fig. 6b). The opposite trend was seen with *Hb9::Foxp1* MNs (mean±s.e.m.=41.7±3.6% axial versus 58.9±2.8% limb) (Fig. 6a,b). Furthermore, we observed a significant increase in size of the neuromuscular junctions when *Hb9::Foxp1* MNs were cultured with limb muscle, compared with *Hb9::GFP* MNs (Fig. 6c). However, there was no difference in synapse area between the two cell lines when they were cultured with axial muscle.

Last, we performed whole-cell patch clamp recordings of the *Hb9::GFP* and *Hb9::Foxp1* ESC-derived MNs to assess whether they displayed any differences in functional maturation (Fig. 6d,e). Both *Hb9::GFP* and *Hb9::Foxp1* MNs robustly fired multiple action potentials (APs) on depolarizing current injection as expected for mature MNs, without any disparities in AP frequency or amplitude (mean±s.e.m.=63±1.5 mV *Hb9::GFP*; 60±2.5 mV *Hb9::Foxp1*). These results indicate that Foxp1 misexpression in ESC-derived MNs produces functionally mature MNs with enhanced capacity to innervate limb muscles *in vitro*.

### Transplanted Foxp1-programmed MNs innervate limb muscles

**Figure 7:**
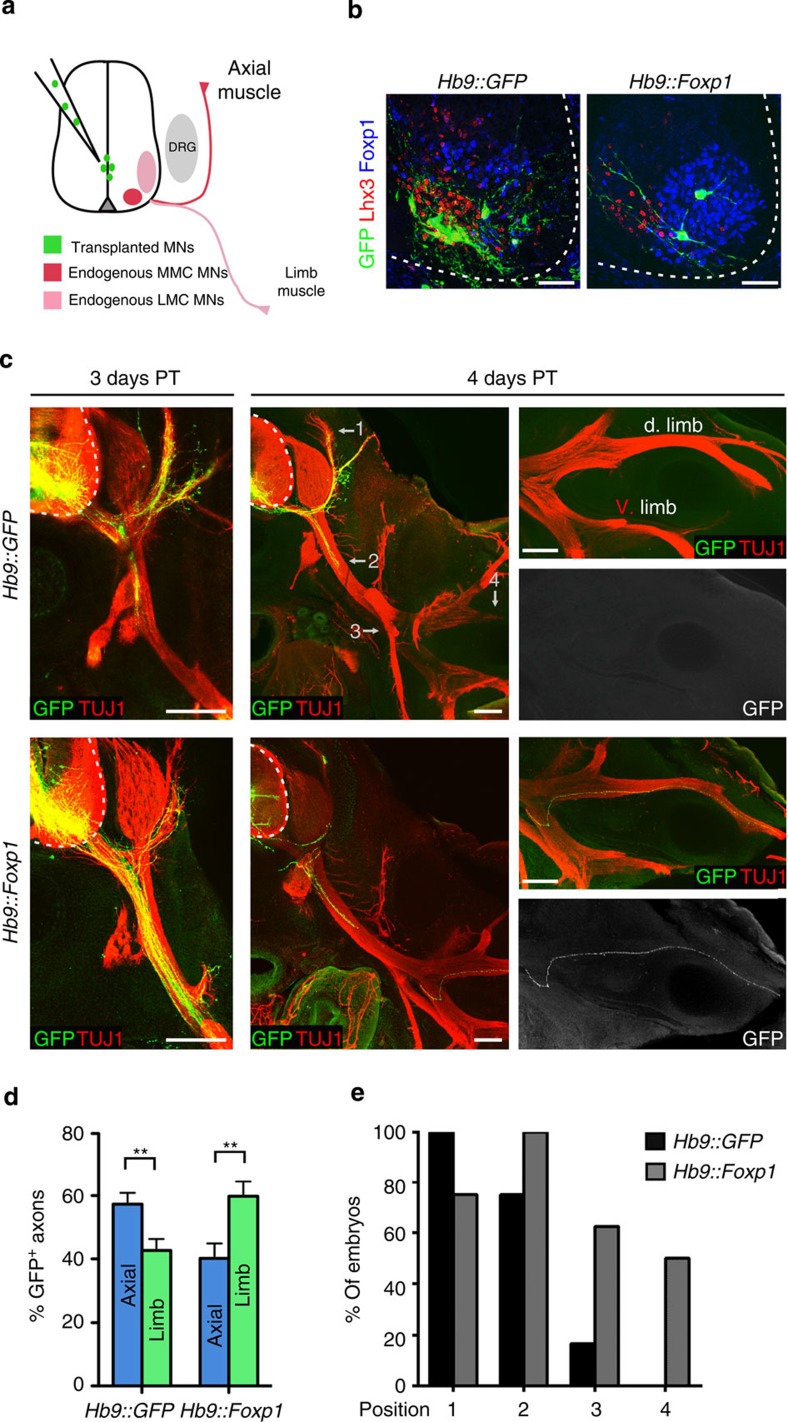
*Hb9::Foxp1* MNs innervate distal limb muscles *in vivo*. (**a**) Transplantation scheme. DRG, dorsal root ganglion. (**b**) Transverse sections of the ventral horn chick spinal cords showing GFP^+^ transplanted MNs. *Hb9::GFP* MNs settled at a medial position associated with endogenous Lhx3^+^ MMC MNs. *Hb9::Foxp1* MNs settled at lateral positions associated with endogenous Foxp1^+^ LMC MNs. Scale bars, 50 μm. (**c**) Transverse vibratome sections of transplantations. *Hb9::GFP* ESC-derived MNs projected axons towards dorsal axial muscles both 3 and 4 days post transplantation (PT). *Hb9::GFP* axons that projected laterally did not extend into the limb. The majority of *Hb9::Foxp1* ESC-derived MNs projected axons laterally towards the limbs 3 days PT and limb projections were maintained at 4 days PT. ‘d. limb’=dorsal limb. ‘v. limb’=ventral limb. ‘DRG’=dorsal root ganglion. Scale bars, 200 μm. (**d**) Quantification of the percentage of transplanted GFP^+^ MN axons that projected towards axial and limb muscles at 3 days PT (mean±s.e.m.; *n*=10 transplants per cell line; Student’s *t*-test). **P=0.008. (**e**) Quantification of transplanted GFP^+^ axons at 4 days PT. Graph shows the percentage of chick embryos that had GFP^+^ axons at positions 1–4. Position 1=axial muscle. Position 2=halfway to brachial plexus. Position 3=brachial plexus. Position 4=distal limb (these are marked in **c**). *n*=8 transplants per cell line.

*Hb9::GFP* MNs have been demonstrated to preferentially project axons towards axial muscle on transplantation into developing chick embryos^6^. A more recent study found that spontaneously differentiated mouse ESC-derived MNs could project axons towards limb muscles on transplantation, but did not show whether they innervated and formed synapses with the limb muscles^19^. We injected GFP^+^ EBs generated from *Hb9::GFP* and *Hb9::Foxp1* ESCs into the neural tube of st.14–17 chick embryos at brachial regions and collected the embryos 3–5 days later (Fig. 7a). First, we confirmed that the molecular profile of the MNs did not change on transplantation. The majority of transplanted *Hb9::GFP* ESC-derived MNs expressed Lhx3 and Hoxa5, despite injection into Hoxc8^+^ brachial regions of the chick neural tube (Supplementary Fig. 6a,b). In contrast, transplanted *Hb9::Foxp1* MNs contained a mixture of Lhx3^+^ (35±6%), Lhx1^+^/Foxp1^+^ (30.6±0.1%) and Foxp1^+^ only (36.6±1.8%) cells, which matched the distribution of MN subtypes characterized *in vitro* (Supplementary Fig. 6a,b). Transplanted *Hb9::GFP* MNs that migrated into the ventral horn of the spinal cord typically settled in a medial position where endogenous MMC MNs are located, while transplanted *Hb9::Foxp1* MNs migrated more laterally towards endogenous LMC MNs (Fig. 7b).

We first analysed axon projections 3 days post transplantation, when both endogenous and transplanted MN axons have reached their target muscles (Fig. 7c). Control *Hb9::GFP* transplants contained more GFP^+^ axons projecting dorsally towards axial muscles than laterally towards limb muscles (mean±s.e.m.=57.3±3.6% axial versus 42.7±3.6% limb). By comparison, *Hb9::Foxp1* transplants showed significantly more GFP^+^ axons projecting laterally towards limb muscles (mean±s.e.m.=40.2±4.7% axial versus 59.8±4.7% limb) (Fig. 7d). At later stages in development, neural circuit refinement occurs as inappropriate or non-functional connections are removed by axon pruning and programmed MN cell death^38^. To see whether increased muscle specificity of transplanted ESC-derived MNs could be observed during this process, we analysed embryos 4 days after transplantation. 100% (8/8) of control *Hb9::GFP* transplants maintained their axial muscle-innervating projections, but none (0/8) contained GFP^+^ projections into the forelimbs (Fig. 7c,e). 75% (6/8) of *Hb9::Foxp1* transplants had GFP^+^ axial projections, which is not unexpected since 35% of transplanted *Hb9::Foxp1* MNs expressed Lhx3. However, 50% (4/8) of *Hb9::Foxp1* transplants also contained GFP^+^ limb projections (Fig. 7c,e). Of these limb projections, the majority of axons (65%) innervated the distal dorsal limb, likely due to their robust expression of EphA4, *c-Ret*, and Lhx1, with the remainder extending ventrally.

To determine whether Foxp1^+^ ESC-derived MNs innervate other types of muscle at non-limb levels of the embryo, we transplanted *Hb9::Foxp1* ESC-derived MNs into the thoracic chick neural tube and analysed axon projections 3 and 4 days later. After 3 days, the majority of embryos contained GFP^+^ axon projections to both axial and intercostal muscles, but not the sympathetic ganglia (Supplementary Fig. 6c). One day later, we found that 60% (3/5) of embryos had GFP^+^ axial projections, but 0% (0/5) had distal projections to the intercostals (Supplementary Fig. 6c,d). These results indicate that *Hb9::Foxp1* ESC-derived MNs failed to stably innervate intercostal muscles, and likely underwent axonal dieback and/or cell death.

We next asked if transplanted *Hb9::Foxp1* ESC-derived MNs projected to both axial and limb muscles due to their mixed composition of MMC and LMC MNs. To address this, we injected dextran into either the axial or limb nerve branches of transplanted chick embryos, and analysed the molecular profile of the retrogradely labelled ESC-derived MNs. When dextran was injected into the axial nerve, 100% of labelled *Hb9::GFP* and *Hb9::Foxp1* ESC-derived MNs expressed Lhx3 and 0% expressed Foxp1 (Supplementary Fig. 7a). In contrast, when dextran was injected into the limb nerve, 0% of labelled *Hb9::Foxp1* ESC-derived MNs expressed Lhx3 and 88.9±11.1% expressed Foxp1 (Supplementary Fig. 7b). *Hb9::GFP* ESC-derived MNs were not labelled after limb nerve injections, in agreement with their failure to innervate limb muscles *in vivo*. These results confirm that *Hb9::Foxp1* ESC-derived MNs innervated muscles appropriate for their MMC and LMC molecular identities.

**Figure 8:**
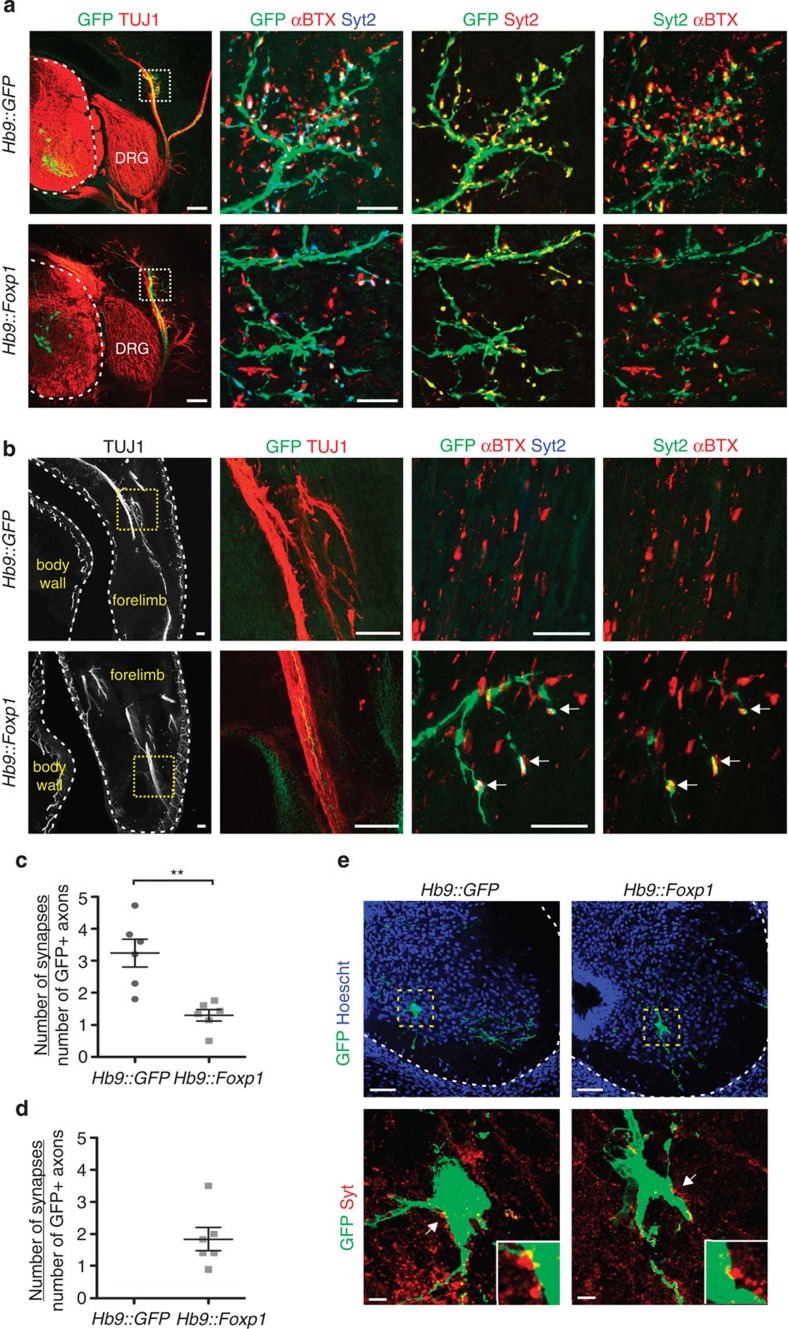
Transplanted ESC-derived MNs form synapses with correct muscles. Analysis of synapse formation 5 days after transplantation of *Hb9::GFP* and *Hb9::Foxp1* ESC-derived MNs. Scale bars, 100 μm for TUJ1 images and 20 μm for αBTX images. (**a**) Transplanted GFP^+^ axons innervating axial muscle. *Hb9::GFP* axons co-localized with a high density of presynaptic (mouse specific Synaptotagmin 2 (Syt2)) and postsynaptic (αBTX) markers. *Hb9::Foxp1* axons displayed fewer synaptic boutons. Boxes represent the areas imaged for synapse markers. DRG, dorsal root ganglion. (**b**) Transplanted GFP^+^ axons innervating limb muscle. *Hb9::GFP* ESC-derived MNs did not innervate and form synapses with limb muscle. *Hb9::Foxp1* axons in the distal limb co-localized with Synaptotagmin 2 and αBTX (arrows). Yellow boxes represent the areas shown in GFP/TUJ1 images where GFP^+^ axons were analysed for synapse markers. (**c**) Quantification of the number of synapses formed by each cell line with axial muscle *in vivo* (mean±s.e.m.; *n*=6 images per cell line; Student’s *t*-test). **P=0.002. (**d**) Quantification of the number of synapses formed by each cell line with limb muscles *in vivo* (mean±s.e.m.; *n*=6 images per cell line; Student’s *t*-test). *Hb9::GFP* control cells showed no synapses in this assay. (**e**) Transplanted ESC-derived MNs in the ventral horns of chick spinal cords (outlined in boxes) had bright Synaptotagmin (Syt)^+^ punctae surrounding their cell body and dendrites. Scale bars, 50 μm in top row and 5 μm in bottom row. Arrows point to regions shown in insets.

We lastly analysed transplanted ESC-derived MNs for presence of synaptic proteins and formation of neuromuscular junctions *in vivo* (Fig. 8). At Hamburger–Hamilton (HH) stages 30–32, transplanted *Hb9::GFP* axons innervated axial muscles, demonstrated by presence of GFP^+^ boutons that contained the presynaptic protein, Synaptotagmin 2 (Fig. 8a). These boutons overlapped with a high number of postsynaptic acetylcholine receptors on chick axial muscles (marked by punctate labelling of Alexa^647^-conjugated αBTX), suggesting formation of neuromuscular junctions. In contrast, *Hb9::Foxp1* axons that innervated axial muscles showed less co-localization of Synaptotagmin 2 and αBTX, indicating fewer synapses (Fig. 8a). To quantify this difference, we counted the number of Synaptotagmin 2^+^/αBTX^+^ synapses normalized to the number of GFP^+^ axons, which showed that *Hb9::GFP* MNs formed significantly more synapses with axial muscle than *Hb9::Foxp1* MNs (Fig. 8c,d). Transplanted *Hb9::Foxp1* MNs by contrast produced many Synaptotagmin 2 and αBTX clusters at the terminals of GFP^+^ axons in dorsal limb regions, a result that was never seen with *Hb9::GFP* MNs (Fig. 8b,d). Finally, using an antibody known to label premotor synaptic terminals in the chick spinal cord^39^, we found bright Synaptotagmin^+^ puncta clustered around the cell bodies and dendrites of both *Hb9::GFP* and *Hb9::Foxp1* MNs (Fig. 8e), suggesting that these transplanted cells are capable of receiving presynaptic inputs from the host spinal cord. Collectively, these data show that Foxp1 misexpression during ESC MN differentiation generates *bona fide* LMC MNs that successfully integrate into the spinal neural circuitry and innervate distal limb muscles *in vivo*, unlike control ESC-derived MNs.

## Discussion

**Figure 9:**
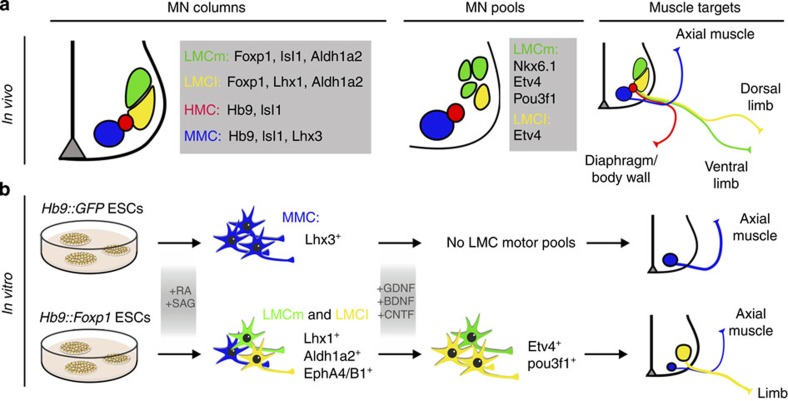
Summary of spinal MN diversity *in vivo* and from ESCs. (**a**) At cervical levels in the developing spinal cord, MNs are segregated into different columns that express different transcription factors and proteins. These columns are further organized into motor pools, or groups of MNs that innervate the same muscle. These different MN subtypes innervate and form synapses with distinct muscles. (**b**) Under standard differentiation conditions with retinoic acid (RA) and smoothened agonist (SAG), the majority of control *Hb9::GFP* ESC-derived MNs expressed Lhx3—a marker of MMC MNs, and did not express markers of LMC MNs. *Hb9::GFP* ESC-derived MNs preferentially projected axons towards and formed synapses with axial muscle both *in vitro* and *in vivo*. Conversely, Foxp1 misexpression resulted in large numbers of *bona fide* LMC MNs that expressed multiple LMCm and LMCl molecular markers and preferentially innervated and formed synapses with limb muscle both *in vitro* and *in vivo*.

MNs are essential for all voluntary and involuntary muscle movements as well as visceral organ functions, illustrated by the catastrophic and often fatal loss of motor control associated with MN disease^8^. Considerable effort has been accordingly directed towards generating MNs from stem cells for use in modelling MN disease pathologies, and potentially as cellular therapies^1^,^2^,^3^,^4^,^5^. In most stem cell studies, little attention is given to the fact that MNs *in vivo* are not a single cell type, but rather a collection of functionally distinct neurons. In the developing spinal cord, MNs are segregated into topographically organized columns and pools along rostrocaudal, mediolateral and dorsoventral axes, largely controlled by the expression of unique transcription factor combinations (Fig. 9a)^7^,^8^,^9^,^10^,^11^,^12^,^13^. This organization allows MNs to correctly innervate different body muscles and receive appropriate presynaptic inputs from sensory neurons, spinal interneurons and descending pathways needed to form stable and functional motor circuits^40^. In agreement with previous reports^6^,^19^, we have found that differentiation of mouse and human ESCs with RA and SAG, a protocol that is widely used in MN differentiation studies, predominantly produces MMC MNs that preferentially innervate and form synapses with axial but not limb muscles (Fig. 9b). Our results show that the molecular and functional diversity of these stem cell-derived MNs can be expanded through the programmed expression of Foxp1, which transforms newly born MNs towards an LMC fate (Fig. 9b). Importantly, *Hb9::Foxp1* ESC-derived MNs innervate and form synapses with distal limb muscles—the defining property of LMC MNs.

One of the current bottlenecks to achieving MN diversity *in vitro* is our limited understanding of how this process occurs *in vivo*, particularly the nature of environmental signals involved. Removal of exogenous growth factors has been shown to broaden the types of MNs generated *in vitro*, but this approach diminishes MN production efficiency to ∼8% of total cells^19^. About ∼38% of spontaneously generated ESC-derived MNs express Foxp1 (that is, ∼3% of the total cells), and only a portion of these cells express LMC motor pool markers such as Etv4 and Pou3f1 (∼18% and ∼9% of Foxp1^+^ MNs, respectively)^19^. Moreover, few Lhx1^+^ LMCl MNs are generated under these conditions even after the addition of RA (∼15% of Foxp1^+^ MNs, that is, <0.5% of total cells)^19^. This markedly contrasts with our results, where 30–40% of the total cells become MNs, over half of which express high levels of Foxp1 and adopt an LMC identity (that is, 15–20% of total cells). It was recently observed that addition of two types of Shh agonists (Purmorphamine and SAG) in addition to RA, results in accelerated generation of Foxp1^+^ MNs from human pluripotent stem cells^41^. ∼About 30% of the cells become MNs, of which ∼65% express Foxp1 (that is, ∼20% of total cells). Interestingly, only 1–5% of these Foxp1^+^ MNs express Lhx1 (ref. 41). It remains to be seen whether these hESC-derived MNs also express LMC motor pool markers and preferentially innervate limb muscles.

It is notable that both of these previously described protocols for generating Foxp1^+^ ESC-derived MNs favour LMCm production. This contrasts with *Hb9::Foxp1* ESCs, which preferentially form LMCl rather than LMCm MNs, reflected in high levels of Lhx1, Etv4, EphA4 and *c-Ret* expression. As many as 40% of *Hb9::Foxp1* MNs expressed Lhx1, and 32% of total MNs expressed Etv4—both significant increases over previous methods. These MNs also displayed dramatically altered axon projections *in vitro* and *in vivo*, with both assays showing a preference for dorsal limb muscle over axial muscle. LMCl MNs were also better restored to wild-type levels than LMCm or HMC MNs in *Hb9::Foxp1; Foxp1*^*−/−*^ embryos. While the bias seen *in vitro* could be partially explained by the inclusion of RA in the MN differentiation procedure, as RA is known to promote LMCl formation^22^, it doesn’t easily account for the bias seen *in vivo*. Another factor that could potentially influence LMC subdivision is the differential activity of the Hb9 promoter used to drive ectopic Foxp1 expression in different MN subtypes. While Hb9 is expressed by all somatic MNs, it is notably elevated in LMCl MNs and low in LMCm MNs^12^,^13^,^15^,^42^,^43^. Consequently, LMCl cells may contain more ectopically expressed Foxp1 than their LMCm counterparts, which could increase the efficiency and/or completeness of their transformation.

Despite these predilections, *Hb9::Foxp1* ESC-derived MNs still produced substantial numbers of Foxp1^+^/Lhx1^*−*^/Pou3f1^+^ MNs, which likely represent an LMCm-associated motor pool^25^, and were also capable of innervating ventral limb muscles on transplantation. We further found that Foxp1 misexpression did not alter Hox protein expression, which agrees with previous reports of Foxp1 acting downstream or in combination with cervical Hox proteins to direct LMC motor pool formation in the embryonic spinal cord^12^,^13^,^24^. The signals required for the formation of most cervical LMC motor pools remain unknown; however, seeing as *Foxp1*^*−/−*^ embryos lack all known LMC motor pool markers^12^,^13^, it seems likely that Foxp1 plays a central role in the assignment of pool identities. In this regard, Foxp1 overexpression during ESC MN differentiation could be a useful tool for identifying novel Foxp1 targets in spinal MNs.

In addition to using exogenous signalling molecules to direct the formation of MNs from pluripotent stem cells, others have begun using transcriptional programming approaches to directly generate MNs from ESCs^44^ and fibroblasts^45^. The types of MNs generated from these procedures remain unclear; however, they most likely represent MMC MNs because Lhx3 overexpression is required for MN conversion^44^,^45^. Interestingly, MNs directly converted from fibroblasts cluster separately from embryonic and ESC-derived MNs in microarray analyses^45^, suggesting that directly converted MNs retain some epigenetic differences related to their fibroblast origin. In our study, we present a hybrid approach incorporating both developmental signalling molecules and transcriptional programming to generate LMC MNs efficiently *in vitro*. The advantages of this method are that it maintains normal differentiation efficiencies, could be applied to both mouse and human systems, and can be adapted to produce other types of MNs, such as thoracic and lumbar populations, as long as candidate transcription factors are known. In principle, it should be possible to combine the approach of misexpressing Foxp1 in combination with different Hox proteins to create PGC and lumbar LMC MNs from ESCs, which have never been effectively generated *in vitro*.

Finally, our studies stress the importance of knowing what type of MNs are being produced within the stem cell cultures for the purposes of disease modelling and cellular therapies. Limb-innervating MNs are thought to be among the earliest affected in ALS, while MMC and HMC MNs are principally affected in spinal muscular atrophy^8^,^46^. It is thus possible that *Hb9::Foxp1* ESC-derived MNs might exhibit enhanced sensitivity to ALS-associated mutations, compared with the MMC MNs typically produced from other ESCs. These differences in MN identity are also paramount for the successful use of stem cell-derived MNs in regenerative contexts. Our results show that different classes of ESC-derived MNs exhibit distinct axon growth potentials and preferences in forming synaptic contacts. It is notable that transplanted *Hb9::Foxp1* ESC-derived MNs successfully innervated and formed synapses with limb muscles *in vivo*, an activity that was not seen with control *Hb9::GFP* MNs. These latter cells were nevertheless capable of innervating axial muscles consistent with their MMC identity. These results are reminiscent of experiments in which thoracic spinal cord segments were transplanted into lumbar levels of chick embryos. The transplanted thoracic MNs initially innervated hindlimb muscles, but at later ages, both MNs and muscles degenerated resulting in impaired hindlimb motor functions^47^,^48^. Together, these studies suggest that correct matching of MN and muscle subtypes may be critical for optimal synapse formation and function. This detail is incredibly important in the context of MN diseases—both for modelling disease pathology *in vitro* with MN-muscle co-cultures and for transplantation into the spinal cord to replace damaged cells. It is very likely that generating specific MN subtypes from stem cells and matching them with appropriate and inappropriate muscle types will reveal novel and significant discoveries into MN development and neurodegenerative disease pathologies.

## Methods

### Mouse husbandry

*Hb9::GFP*, *Hb9::Foxp1*, and *Foxp1*^*+/−*^ heterozygous mice were maintained as previously described^13^,^15^,^49^. *Hb9::Foxp1* heterozygous mice were crossed with *Foxp1*^*+/−*^ mice to generate *Hb9::Foxp1; Foxp1*^*+/−*^ mice. These mice were then interbred with *Foxp1*^*+/−*^ mice to produce *Hb9::Foxp1; Foxp1*^*−/−*^ embryos. The *Hb9::Foxp1* transgene contains an IRES-GFP sequence but GFP expression levels were very low in ESC-derived MNs. All mice were maintained and tissue were collected in accordance with guidelines set forth by the UCLA Institutional Animal Care and Use Committee.

### *Hb9::Foxp1* mouse ESC line generation and characterization

Superovulation was induced in *Hb9::GFP* heterozygous female mice by sequential injections of pregnant mare serum gonadotropin (5 IU) followed by human chorionic gonadotropin (50 IU) 48 h later per female. These mice were paired overnight with *Hb9::Foxp1* transgenic male mice. E0.5 pre-implantation embryos were isolated the next morning and incubated in hyaluronidase (0.3 mg ml^*−*1^; Sigma) until cumulus cells were removed from the embryos. About 24 embryos were collected in total and cultured in KSOM media (Millipore) until the blastocyst stage^50^. About 15 out of 24 embryos developed into blastocysts and were plated on mitomycin-treated mouse embryonic fibroblasts (MEFs) in mouse ESC media (Dulbecco's Modified Eagle's Medium/ (DMEM) high glucose, 10% ES-qualified FBS, 1% penicillin/streptomycin, 1% glutamax, 1% nonessential amino acids, Primocin (50 μg ml^*−*1^; Invivogen), 2-mercaptoethanol (560 nM) and Leukaemia Inhibitory Factor (ESGRO; Millipore)) plus MEK1 inhibitor (50 μM; PD98059; Cell Signaling Technologies). Except as noted, media components were obtained from Invitrogen. About 4 days after plating, 12 blastocysts had hatched and attached to MEFs. Attached inner cell masses were monitored and passaged after 3–4 days of growth. MEK1 inhibitor was removed from the media after the appearance of ES colonies. Mouse ESC colonies appeared within 1 week and were expanded on MEFs until confluent^51^. Ten final mouse ESC lines were genotyped for presence of the two transgenes. Four lines contained both the *Hb9::Foxp1* and *Hb9::GFP* transgenes and three lines contained only the *Hb9::GFP* transgene. All mouse ESCs were maintained in mouse ESC media on MEFs.

### Mouse ESC differentiation to MNs

HBG3 *Hb9::EGFP* mouse ESCs (a gift from H. Wichterle, Columbia University) and newly generated *Hb9::GFP* and *Hb9::Foxp1*; *Hb9::GFP* mouse ESCs were maintained and differentiated into MNs as previously described^16^,^52^. Briefly, mouse ESCs were first plated on gelatin to remove MEFs prior to differentiation, and then plated in 60 mm bacterial Petri dishes in core MN medium to induce EB formation. Core MN medium consisted of a 1:1 mixture of DMEM/F12 and Neurobasal Medium supplemented with 10% knockout serum replacement, 1% Glutamax, 2-mercaptoethanol (560 nM), 1% penicillin/streptomycin and Primocin. Except as noted, media components were obtained from Invitrogen. About 2 days later, N2 supplement (1 × , Invitrogen), RA (1 μM; Sigma), and SAG (1 μM; Calbiochem) were added to the EBs. After 5–6 days, the EBs were either dissociated and plated on matrigel-coated coverslips for immunostaining or used for transplantation and muscle assays. For culturing the MNs past day 6, RA and SAG were removed from the medium and replaced with GDNF (10 ng ml^*−*1^; Peprotech), Brain-Derived Neurotrophic Factor (10 ng ml^*−*1^; Peprotech), and Ciliary Neurotrophic Factor (10 ng ml^*−*1^; Peprotech).

### Cell sorting of stem cell-derived MNs and RNA isolation

*Hb9::GFP* and *Hb9::Foxp1* EBs were collected 5–7 days after RA and SAG addition. EBs were dissociated using ice-cold Trypsin-EDTA (0.25%; Invitrogen) for 6 min at room temperature, followed by trituration in L-15 Leibovitz media (Thermo Scientific)^53^. The dissociated cells were pipetted through 40 μm cell strainers and live GFP^+^ MNs (identified as lacking staining for 7AAD) were sorted and collected using a BD ARIA II FACS machine in the UCLA Broad Stem cell center core facility. Total RNA was isolated from GFP^+^ MNs using an RNAeasy MiniKit (Qiagen).

### Quantitative PCR

Quantitative PCR analysis of MN populations was performed as previously described^21^. The following primer pairs were used: mouse *Foxp1 3*′*UTR*, forward 5′-cagccacgaaagaaacagaag-3′ and reverse 5′-ggtcctggtcacctgattata-3′; mouse *Aldh1a2*, forward 5′-catggtatcctccgcaatg-3′ and reverse 5′-gcgcatttaaggcattgtaac-3′; mouse *EphA4*, forward 5′-ctgagccagtaccgaataga-3′ and reverse 5′-gagggcgaagacaaagtaaa-3′; mouse *EphB1*, forward 5′-gaagaaaggaaggaggtggaa-3′ and reverse 5′-agggaggagtaagaaaggagaa-3′; mouse *c-Ret*, forward 5′-ctgactgaggtgagagaacaag-3′ and reverse 5′-acacgtaccatagcagcataaa-3′; mouse *c-Met*, forward 5′-tcagccatcccaatgttctc-3′ and reverse 5′-cgcagatctccatgcttcata-3′; mouse *Fgfr1*, forward 5′-aggttagccagagggtagaa-3′and reverse 5′-aggagaggaatatgaggagtagg-3′; mouse *β-Actin*, forward 5′-gccaaccgtgaaaagatgac-3′ and reverse 5′-gaggcatacagggacagcac-3′. Relative mRNA expression levels were determined by normalizing crossing points for each gene to *β-Actin* and calculating fold changes of Foxp1 misexpression over control *Hb9::GFP* MNs.

### Immunostaining

All embryos and cell cultures were fixed in 4% paraformaldehyde (Fisher). Antibody staining of cryosections and vibratome sections were performed as previously described^13^,^21^,^54^. Fixed adherent cell cultures were washed in block solution (PB, 1% heat-inactivated horse serum, 0.1% Triton X-100) for 10 min, followed by incubation with primary antibodies overnight at 4 °C. The following primary antibodies were used: guinea pig anti-Aldh1a2 (1:20,000)^55^, goat anti-ChAT (1:200; Millipore AB144P)^52^, rabbit anti-EphA4 (1:500; Santa Cruz Sc-291)^13^, rabbit anti-Etv4 (1:1,000)^56^, guinea pig anti-Foxp1 (1:16,000)^13^, sheep anti-GFP (1:800; AbD Serotec 4745-1051), rabbit anti-GFP (1:3,000; Life Technologies A6455)^54^, chick anti-GFP (1:1,000; Aves Labs GFP-1020), rabbit anti-Hb9 (1:8,000)^42^, guinea pig anti-Hoxa5 (1:8,000)^25^, goat anti-Hoxa9 (1:1,000; Santa Cruz SC-17155)^13^, rabbit anti-Hoxc6 (1:1,000; Santa Cruz SC-66925)^13^, mouse anti-Hoxc8 (1:2,000; Covance MMS-266R)^13^, goat anti-Isl1 (1:8,000, R&D Systems AF1837)^13^, mouse anti-Isl1/2 (39.4D5; 1:100; Developmental Studies Hybridoma Bank)^57^, mouse anti-Lhx1 (4F2; 1:100; Developmental Studies Hybridoma Bank)^58^, rabbit anti-Lhx3 (1:1,000; Abcam Ab14555), mouse anti-Lhx3 (67.4E12; 1:100; Developmental Studies Hybridoma Bank)^57^, mouse anti-myosin heavy chain (MF20; 1:100; Developmental Studies Hybridoma Bank)^59^, rabbit anti-Nkx6.1 (1:2,500)^60^; rabbit anti-nNOS (1:10,000; ImmunoStar 24287)^13^, rabbit anti-Pou3f1 (1:2,000)^61^; mouse anti-Pou5f1 (1:1,000; Santa Cruz SC-5279), goat anti-Sox2 (1:2,000, Santa Cruz SC-17320)^21^,^54^, mouse anti-Synaptotagmin (mAb 30; 1:100; Developmental Studies Hybridoma Bank)^62^, mouse anti-Synaptotagmin 2 (znp-1; 1:100; Developmental Studies Hybridoma Bank)^63^,^64^, rabbit anti-βIII Tubulin (1:3,000; Covance MRB-435P) and mouse anti-βIII Tubulin (1:3,000; Covance MMS-435P). Monoclonal antibodies obtained from the DSHB were developed under the auspices of the NICHD and maintained by the University of Iowa, Department of Biology, Iowa City, IA 52242. Cells were washed and then incubated with Alexa^488^-, FITC-, Cy3-, Cy5- and DyLight^649^-conjugated secondary antibodies (Jackson Immunoresearch) for 1 h at room temperature the following day, and coverslips were mounted with Prolong Gold (Invitrogen). Representative images shown for antibody staining were successfully repeated at least 2–3 times.

### Image analysis

DIC images were collected using a Zeiss Axioobserver microscope equipped with the Apotome optical imaging system. Fluorescent images were collected using either a Zeiss LSM5 Exciter or Zeiss LSM780 confocal imaging system. Images were processed using Adobe Photoshop and CorelDraw Software. Cell number quantification was performed using the NIH ImageJ software suite with an ITCN add-in for automated cell counting.

### Muscle and EB co-cultures

*Hb9::GFP* and *Hb9::Foxp1* ESC-derived EBs with high levels of GFP expression were manually collected 5–6 days after RA/SAG addition. Axial, forelimb, dorsal hindlimb and ventral hindlimb muscles were dissected from HH stage 35 chick embryos. For adherent muscle co-cultures: dissected muscles were dissociated and plated onto matrigel-coated glass bottom dishes (MatTek Corporation). *Hb9::GFP* and *Hb9::Foxp1* EBs were plated on top of the dissociated muscle and cultured for 3 days. Synapse formation was assayed using Alexa^647^-conjugated αBTX (1:300; Invitrogen). For three-dimensional collagen cultures, muscle explants were placed on either side of a single EB in a collagen bed (rat tail collagen IV, Calbiochem)^65^ and cultured for 24 h. About ∼80% of muscle explants showed >70% myosin heavy chain positive staining and ∼90% of explants showed axon growth. Data was extracted from this latter group. In both assays, muscle and MN co-cultures were incubated in core MN medium with N2 supplement and heparin (10 μg ml^*−*1^; Sigma).

### Electrophysiology

*Hb9::GFP* and *Hb9::Foxp1* EBs were dissociated using papain (0.5 U ml^*−*1^; Worthington) and plated at low density on Matrigel-coated 35 mm culture dishes (1.2 × 10^4^ cells per dish) in core MN medium with neurotrophic factors and cultured for an additional 2–3 days. Recordings were made from GFP^+^ cells using standard whole-cell, current-clamp techniques at 20–22 °C. The recording bath solution contained: NaCl (120 mM), KCl (1.9 mM), KH_2_PO_4_ (1.2 mM), Na-bicarbonate (20 mM), CaCl_2_ (2.2 mM), MgCl_2_ (1.4 mM), D-Glucose (10 mM) and Hepes (7.5 mM). Patch pipettes (resistance 3–5 MΩ) were filled with internal recording solution containing: K-gluconate (140 mM), Hepes (10 mM), EGTA (1 mM), Mg-ATP (4 mM) and Na-GTP (0.3 mM). The pH of both solutions was adjusted to 7.2. Cells with resting potentials <−30 mV were discarded. Patched MNs (maintained in current-clamp mode at approximately −70 mV) were stimulated with 0.1 nA depolarizing current pulses of 250 ms duration. For each MN, this stimulus pulse was applied at least three times (with intervals ≥30 s). Only data from cells in which the long stimulus pulse elicited the same number of APs were included in this study. Data were collected using Axopatch 2B patch clamp amplifiers with 4-pole Bessel filtering at 5 kHz. Signals were digitized, stored and analysed using pClamp software (Axon Instruments). AP amplitude was measured as the voltage change from the threshold to the peak of the first AP in a train.

### Chick transplantations

Fertilized chicken eggs (McIntyre Poultry and Fertile Eggs) were incubated at 38 °C for ∼60 h to HH stage 14–17 prior to use. *Hb9::GFP* and *Hb9::Foxp1* ESC-derived MNs were transplanted into the lumen of the spinal cord, as described previously^53^. Briefly, GFP^+^ EBs were manually collected 5–6 days after RA and SAG addition and cut into 4–6 pieces. A glass needle was inserted through a small nick in the neural tube of the embryo and a small portion of the neural tube was removed at brachial or thoracic levels. GFP^+^ EBs were injected into this lesion site. Embryos were allowed to develop for 2–5 days post transplantation and then collected, fixed and prepared for cryosections or vibratome sections.

### Retrograde labelling of transplanted MNs

*Hb9::GFP* and *Hb9::Foxp1* ESC-derived MNs were transplanted into the chick spinal cord at brachial levels. About 3 days later embryos with GFP^+^ cells were identified and used for retrograde tracing experiments. Dextran (Biotinylated 3,000 MW; 10 mg ml^*−*1^; Invitrogen) was injected into the axial or limb nerve branches. Embryos were cultured at 30 °C for 6–7 h, fixed and prepared for cryosections.

### Human ESC culture and differentiation to MNs

All human ESC experiments were conducted with prior approval from the UCLA Embryonic Stem Cell Research Oversight (ESCRO) Committee. H9 human ESCs (Madison, Wisconsin) were maintained on irradiated MEFs in human ESC media (DMEM/F12, 20% knockout serum replacement, 1% nonessential amino acids, 1% glutamax, 0.1 mM 2-mercaptoethanol and 10 ng ml^*−*1^ of basic fibroblast growth factor). Human ESCs were differentiated to MNs as previously described^14^. RA was added to the human ESC-derived neural progenitors after 10 days of differentiation. Neural rosettes were manually isolated and transferred into neural differentiation media with 0.1 μM RA and 1 μM SAG. After 28 days, human ESC-derived MN progenitors were cultured in human MN maturation media with 0.1 μM RA, 50 nM SAG, cyclic AMP (0.1 μM; Sigma), ascorbic acid (200 ng ml^*−*1^; Sigma), insulin growth factor 1 (10 ng ml^*−*1^; Peprotech) and NFs (GDNF, BDNF and CNTF; all 10 ng ml^*−*1^; Peprotech)). For immunostaining, hESC-derived MNs were lightly dissociated using 20% TrypLE (Invitrogen) and plated on matrigel-coated glass coverslips in MN maturation media.

### Lentiviral transduction of human ESC-derived MNs

The coding sequence of human *FOXP1 (*isoform 1) was PCR amplified from the plasmid template HsCD00297105 (PlasmID Database, Dana Farber/Harvard Cancer Center DNA Resource Core), and then cloned into the FUCRW lentiviral vector^66^. Control FUCRW (RFP) and FUCRW–FOXP1 lentiviruses were generated using standard third generation protocols, and concentrated viral aliquots were stored at −80 °C. Newly born human ESC-derived MNs (∼35 days after start of differentiation) were infected overnight with the concentrated lentiviruses. RFP^+^ cells were identified 3–5 days after infection. About 1–2 weeks after infection, transduced human ESC-derived MNs were fixed in 4% paraformaldehyde for 10 min at room temperature before being processed for immunostaining.

### Quantification

For analysis of MN populations in E12.5 mouse embryos: mean±s.e.m. values were calculated by pooling at least three sections at both cervical and thoracic levels, collected from at least three embryos of each genotype. For analysis of ESC-derived MNs: 3–5 field images containing ∼100 cells per field were counted for each antibody stain per differentiation batch. Values from three separate differentiation batches were averaged to generate mean±s.e.m. values shown in the figures.

### Statistics

No statistical methods were used to predetermine sample sizes, but our sample sizes are similar to those reported in previous publications^13^,^19^. All quantification of *in vitro* MN-muscle co-cultures and explants was performed blind. Blinding of other experiments and randomization were not performed. Data were not tested for normality and compared using either the Student’s *t*-test, a paired two-tail *t*-test, or one or two-way analysis of variance with Bonferroni adjustments for multiple comparisons. Variance was similar between groups being compared. The test used for each experiment is noted in the figure legends. All statistics and graphs were generated using Prism6 Graphpad software.

## Additional information

**How to cite this article:** Adams, K. L. *et al*. Foxp1-mediated programming of limb-innervating motor neurons from mouse and human embryonic stem cells. *Nat. Commun*. 6:6778 doi: 10.1038/ncomms7778 (2015).

## Supplementary Material

Supplementary Information Supplementary Figures 1-7
