# Supplementary material for: Foxp1-mediated programming of limb-innervating motor neurons from mouse and human embryonic stem cells
Source: Nat Commun. 2015 Apr 14;6:6778. doi: 10.1038/ncomms7778 (PMC4397664; doi:10.1038/ncomms7778)
Supplement: Supplementary Information — Supplementary Figures 1-7 [file ncomms7778-s1.pdf]

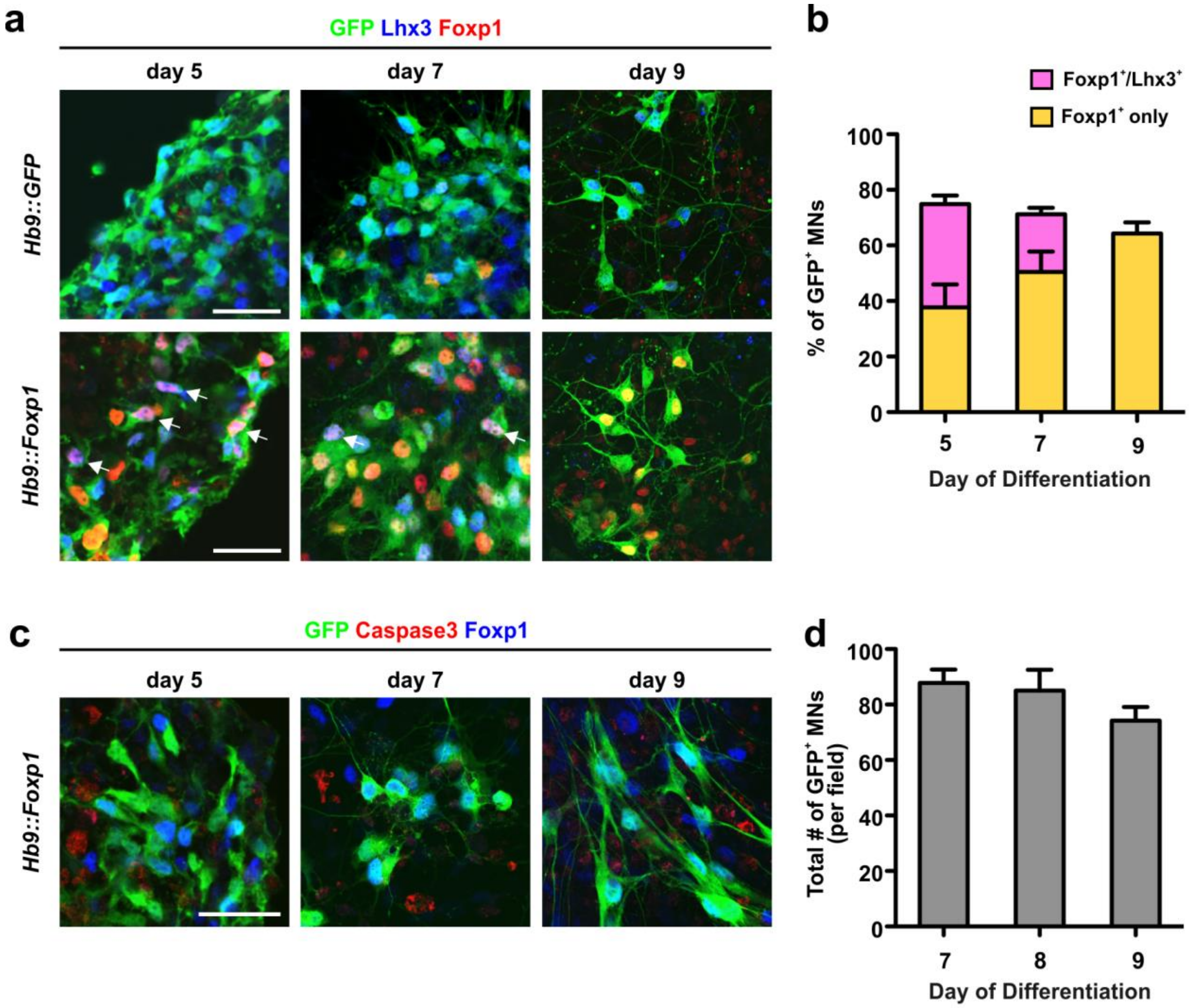

Supplementary Figure 1 | Lhx3^{+}/Foxp1^{+} *Hb9::Foxp1* MNs resolve over time in culture. *Hb9::GFP* and *Hb9::Foxp1* ESC-derived MNs were analyzed by antibody staining for the presence of Lhx3^{+}/Foxp1^{+} MNs at three time-points after RA/SAG addition. (**a**) *Hb9::GFP* ESC-derived MNs maintained expression of Lhx3 and did not express Foxp1. A large number of *Hb9::Foxp1* ESC-derived MNs co-expressed Lhx3 and Foxp1 early in differentiation (arrows), but by day 9, Lhx3 expression had turned off in Foxp1^{+} MNs. Scale bars = 50 µm. (**b**) Quantification of the percentage of Lhx3^{+}/Foxp1^{+} and Foxp1^{+} only MNs generated from *Hb9::Foxp1* ESCs over time (mean ± s.e.m.; *n* = 5-7 images per day, 520 total MNs for day 5, 478 total MNs for day 7, 264 total MNs for day 9). (**c**) *Hb9::Foxp1* ESC-derived MNs did not express activated Caspase3 during days 5 to 9 of differentiation. Scale bar = 50 µm. (**d**) Quantification of the total number of *Hb9::Foxp1* ESC-derived MNs per field of view from days 7 to 9 of differentiation (mean ± s.e.m.; *n* = 3 independent experiments, 912 total MNs).

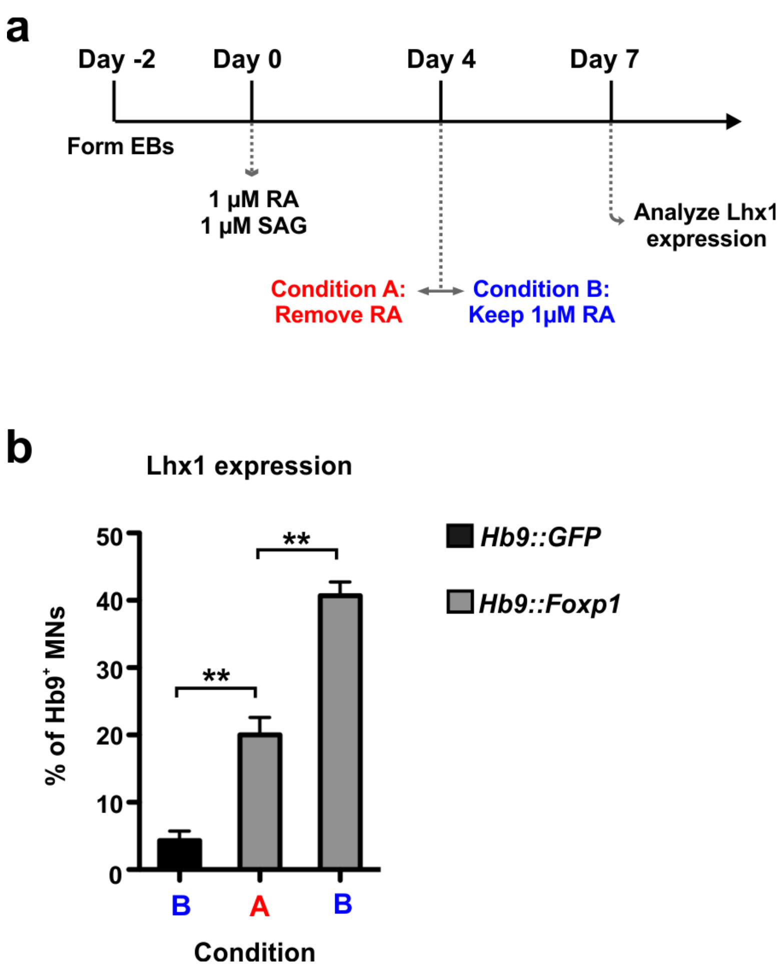

Supplementary Figure 2 | RA levels alter the number of Lhx1+ *Hb9::Foxp1* MNs. (**a**) Schematic diagram of differentiation protocol used to analyze effects of RA concentration. ESC-derived cultures either received RA only up to day 4 (Condition A) or throughout the entire protocol (Condition B). All cultures were analyzed on day 7. (**b**) Quantification of the percentage of Lhx1+ LMCl MNs generated by *Hb9::GFP* and *Hb9::Foxp1* ESC-derived MNs in conditions A and B (mean ± s.e.m.; n = 3 independent experiments, 387 *Hb9::GFP* and 1106 *Hb9::Foxp1* MNs total; one-way ANOVA with Bonferroni adjustments). Left to right: **P = 0.0051 and **P = 0.0014.

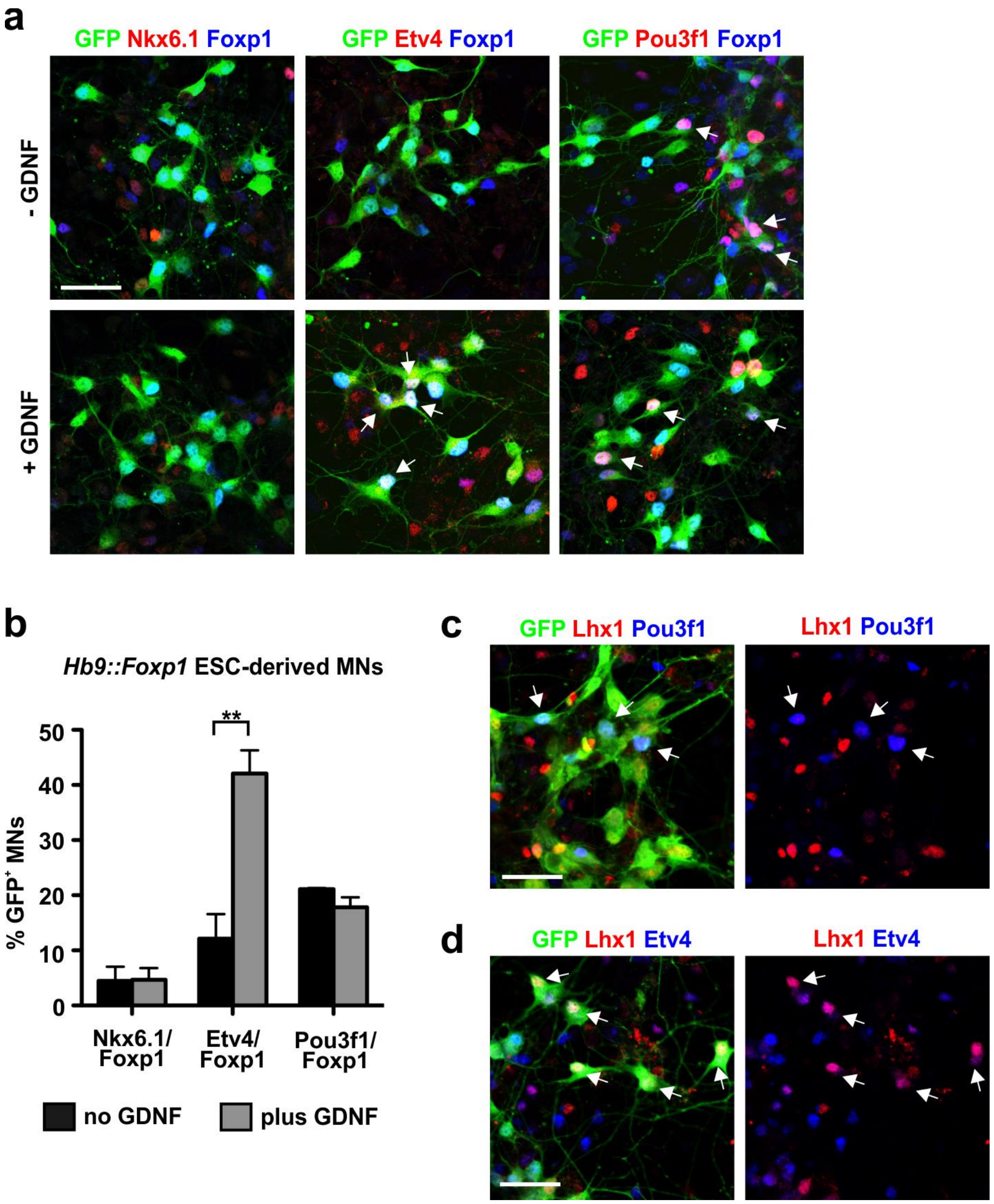

Supplementary Figure 3 | Etv4, but not Pou3f1, expression depends on GDNF exposure. (**a**) In the absence of GDNF (-GDNF) *Hb9::Foxp1* MNs expressed low levels of Etv4, but maintained expression of Pou3f1 (arrows). In the presence of GDNF (+GDNF), *Hb9::Foxp1* MNs expressed high levels of both Etv4 and Pou3f1 (arrows). Foxp1$^+$ MNs did not express Nkx6.1 in either condition. Scale bars = 50 µm. (**b**) Quantification of the percentage of GFP$^+$ MNs that expressed cervical LMC motor pool markers in the absence and presence of GDNF (mean ± s.e.m.; n = 3 independent experiments, 798 total MNs for –GDNF, 1048 total MNs for +GDNF; Student's t-test). **P = 0.008. (**c**) Pou3f1$^+$ *Hb9::Foxp1* MNs did not express Lhx1 (arrows). Scale bar = 50 µm. (**d**) Etv4$^+$ *Hb9::Foxp1* MNs also expressed Lhx1 (arrows). Scale bar = 50 µm.

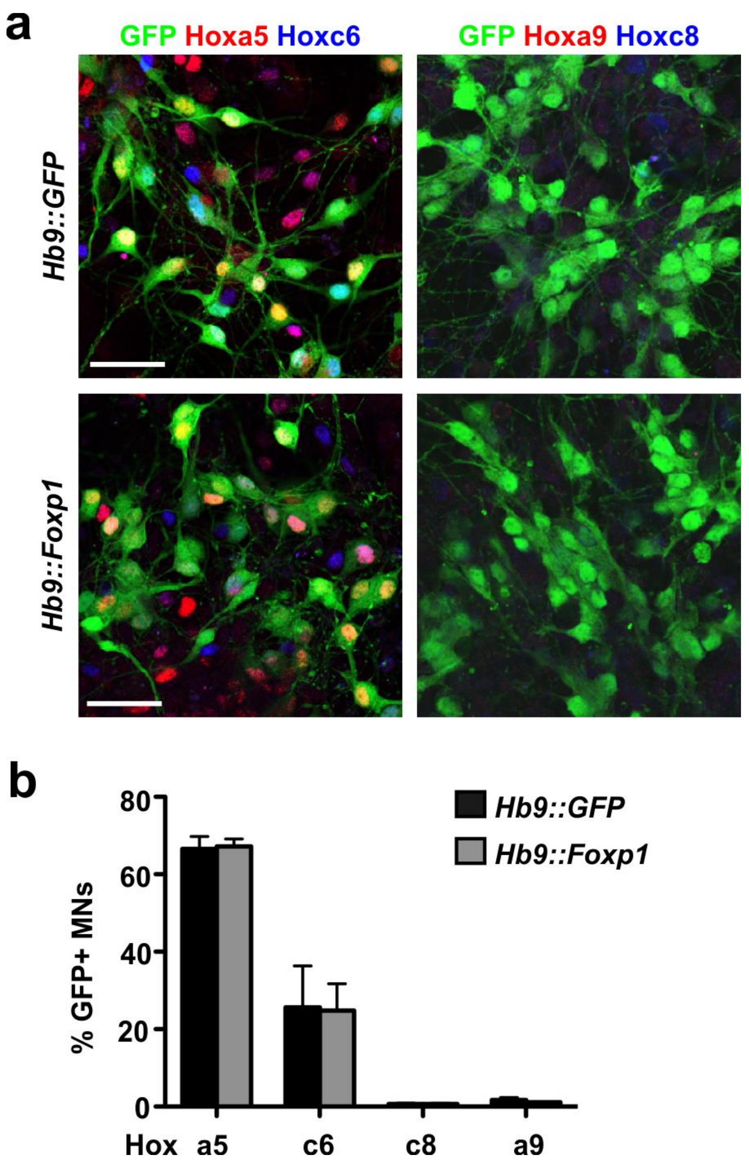

Supplementary Figure 4 | Foxp1 misexpression does not alter MN Hox profiles. (**a**) Both *Hb9::GFP* and *Hb9::Foxp1* ESC-derived MNs expressed rostral forelimb Hox proteins Hoxa5 and Hoxc6, but did not express more caudal Hox proteins Hoxc8 and Hoxa9. Scale bar = 50 μm. (**b**) Quantification of the percentage of GFP^+ MNs that expressed each Hox protein (mean ± s.e.m.; *n* = 3 independent experiments, 1404 *Hb9::GFP* and 891 *Hb9::Foxp1* MNs total).

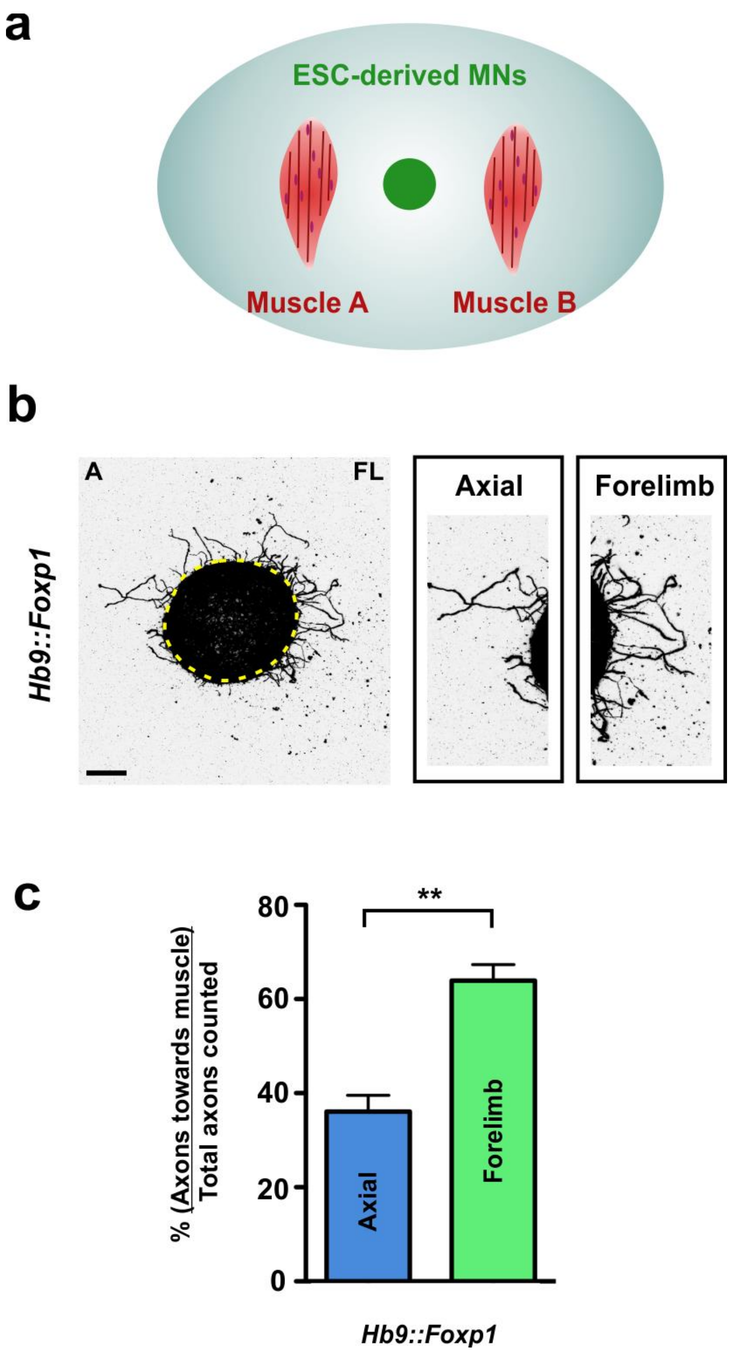

Supplementary Figure 5 | *Hb9::Foxp1* MNs preferentially project to forelimb muscles. (**a**) Schematic diagram of *in vitro* muscle explant assay. (**b**) *Hb9::Foxp1* ESC-derived MNs projected more axons towards forelimb muscle than axial muscle. (**c**) Quantification of *Hb9::Foxp1* axon projections when cultured between axial and forelimb muscle (mean ± s.e.m.; $n = 4$ cultures; Student's t-test). **$P = 0.0012$.

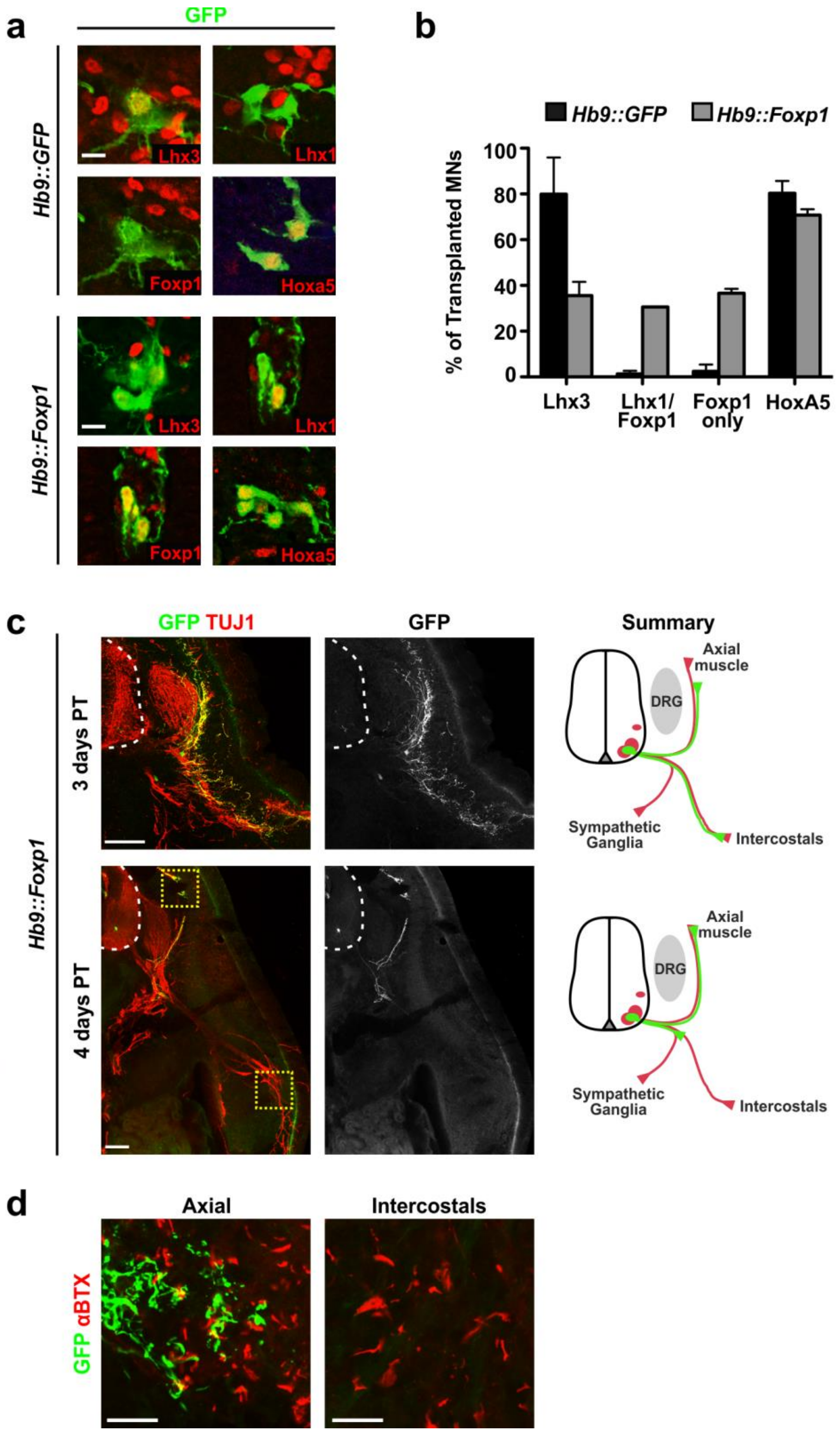

Supplementary Figure 6 | Transplanted ESC-derived MNs fail to innervate intercostals. (**a**) Transplanted *Hb9::GFP* MNs maintained expression of Lhx3 and Hoxa5. *Hb9::Foxp1* transplanted MNs expressed reduced levels of Lhx3 and increased

levels of Foxp1 and Lhx1. All Lhx1$^+$ MNs co-expressed Foxp1. Scale bars = 10 μm. (**b**) Quantification of the percentage of transplanted ESC-derived MNs that expressed different MN subtype markers (mean ± s.e.m.; *n* = 85 *Hb9::GFP* transplanted cells and 79 *Hb9::Foxp1* transplanted cells). (**c**) Transplantation of *Hb9::Foxp1* MNs at thoracic spinal cord regions. GFP$^+$ axons projected towards axial muscle and intercostals at 3 days post transplantation (PT), but only axial projections were maintained at 4 days PT. Scale bars = 200 μm. The arrow and yellow box depict the areas shown in d. (**d**) *Hb9::Foxp1* GFP$^+$ axons co-localized with αBTX$^+$ acetylcholine receptors of axial muscles, but not intercostal muscles. Scale bars = 20 μm.

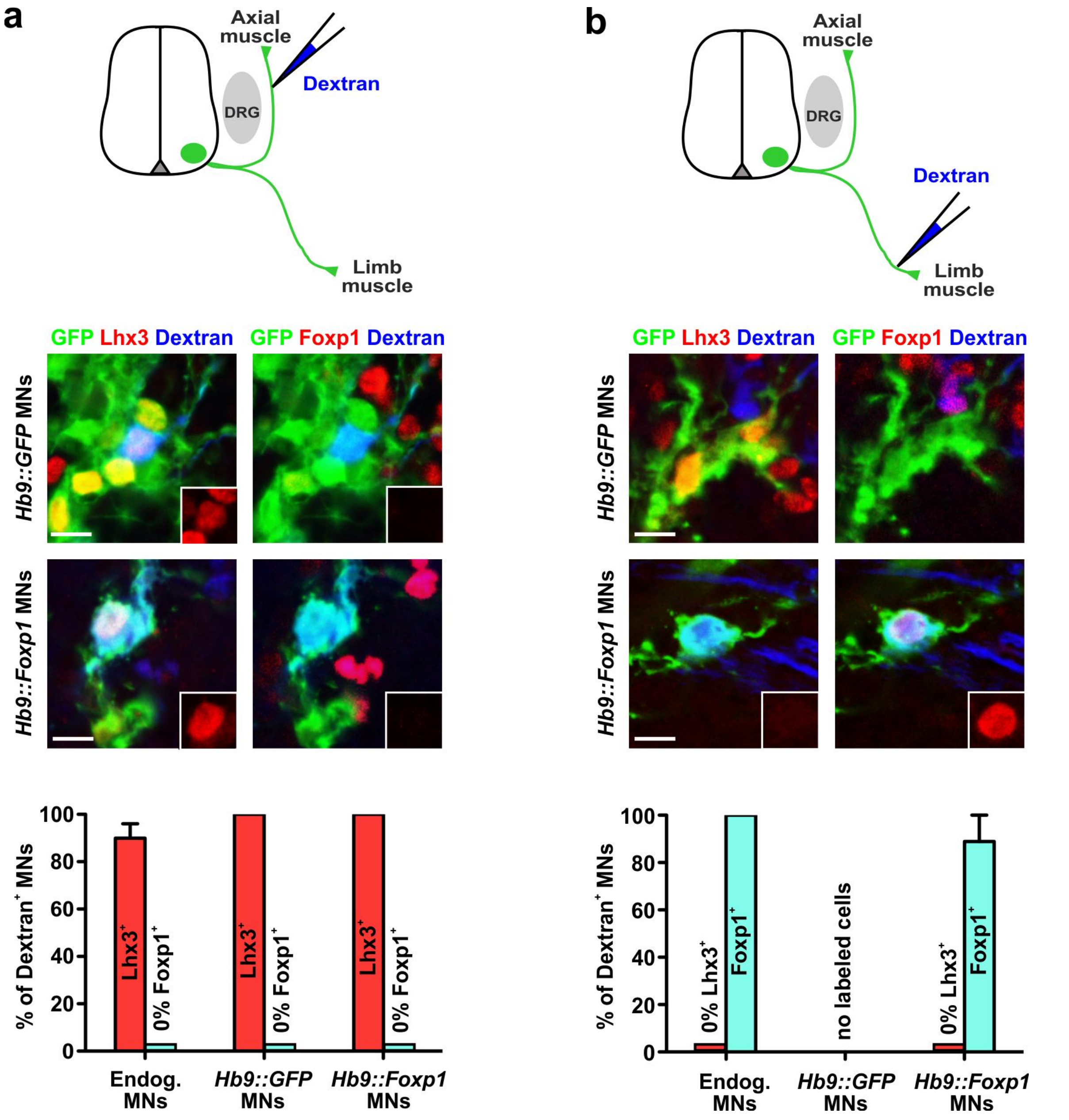

Supplementary Figure 7 | ESC-derived MNs innervate in accordance to their molecular identities. (**a**) Retrograde labeling of MMC MNs by dextran injection into the axial nerve branch. Only $Lhx3^+$ endogenous MMC MNs were backfilled. Labeled transplanted *Hb9::GFP* and *Hb9::Foxp1* MNs (GFP^+/$Dextran^+$) expressed Lhx3 and not Foxp1 (mean ± s.e.m.; *n* = 4-6 embryos). Insets show the red channel for $dextran^+$ cells. Scale bars = 10 μm. (**b**) Retrograde labeling of LMC MNs by dextran injection into the limb nerve branch. Only $Foxp1^+$ endogenous LMC MNs were backfilled. Labeled transplanted *Hb9::Foxp1* MNs expressed Foxp1 and not Lhx3 (mean ± s.e.m.; *n* = 4-6 embryos). Transplanted *Hb9::GFP* MNs were not labeled with dextran. Insets show the red channel for $dextran^+$ cells. Scale bars = 10 μm.
